# Research on Longitudinal Active Collision Avoidance of Autonomous Emergency Braking Pedestrian System (AEB-P)

**DOI:** 10.3390/s19214671

**Published:** 2019-10-28

**Authors:** Wei Yang, Xiang Zhang, Qian Lei, Xin Cheng

**Affiliations:** 1 State Key laboratory of Mechanical Transmission, College of Automotive Engineering, Chongqing University, Chongqing 400044, China; leiqian0929@163.com (Q.L.); cquchengxin@163.com (X.C.); 2 School of Information, Zhejiang University of Finance Economics, Hangzhou 310018, China

**Keywords:** AEB-P system, warning model, upper fuzzy neural network controller, lower PID controller, CarSim and Simulink co-simulation

## Abstract

The AEB-P (Autonomous Emergency Braking Pedestrian) system has the functional requirements of avoiding the pedestrian collision and ensuring the pedestrian’s life safety. By studying relevant theoretical systems, such as TTC (time to collision) and braking safety distance, an AEB-P warning model was established, and the traffic safety level and work area of the AEB-P warning system were defined. The upper-layer fuzzy neural network controller of the AEB-P system was designed, and the BP (backpropagation) neural network was trained by collected pedestrian longitudinal anti-collision braking operation data of experienced drivers. Also, the fuzzy neural network model was optimized by introducing the genetic algorithm. The lower-layer controller of the AEB-P system was designed based on the PID (proportional integral derivative controller) theory, which realizes the conversion of the expected speed reduction to the pressure of a vehicle braking pipeline. The relevant pedestrian test scenarios were set up based on the C-NCAP (China-new car assessment program) test standards. The CarSim and Simulink co-simulation model of the AEB-P system was established, and a multi-condition simulation analysis was performed. The results showed that the proposed control strategy was credible and reliable and could flexibly allocate early warning and braking time according to the change in actual working conditions, to reduce the occurrence of pedestrian collision accidents.

## 1. Introduction

According to the latest statistics from the National Bureau of Statistics, in 2017, in China, there had been 203,000 traffic accidents, in which 63,700 people had lost their lives, and direct economic losses of 1.213 billion yuan had been caused. In 2018, the number of traffic accident-caused deaths in China decreased by 578 compared with 2017, representing a decrease of 0.9% [1]. Among all the traffic accidents, the car collision accidents account for about 70%, most of which are rear-end accidents [2], while the traffic accidents caused by improper human operations account for about 90% [3]. In the rear-end collision accidents, 31% of drivers fail to adopt proper braking measures, 20% of drivers do not brake timely, and 49% of drivers fail to brake fully [4]. The fatality rate of pedestrians in traffic accidents is quite high, and the number of pedestrians killed in traffic accidents accounts for about 25% of the total number of accidents every year [5].

Using car-sensing devices (radars, cameras, sensors, etc.), the automatic emergency braking (AEB) system can recognize the surrounding driving environment [6,7], collect the relevant data, perform dynamic and static identification and tracking functions, and adopt the measures, such as early warning and automatic emergency braking based on data analysis and judgment to avoid a collision or reduce collision damage. The AEB system can effectively improve the driving safety of vehicles, reduce the occurrence of collision traffic accidents [8], and the working intensity of drivers.

In order to accelerate the popularization of the AEB system, the Economic Commission of Europe (ECE) has formulated draft regulations for the AEB systems [9]. According to the Euro-NCAP (new car assessment program) regulations, new cars without an AEB system can difficultly obtain the highest safety rating [10]. In addition, the association also introduced the evaluation standards of the AEB-P pedestrian collision avoidance system in 2016 [11]. The Euro-NCAP research results show that the usage of the AEB system can effectively reduce 27% of collision accidents [12]. Based on a large number of theoretical foundations of the AEB system, the AEB-P system has higher technical requirements for pedestrians in target collision avoidance and has become a research hotspot in the field of automotive active safety.

In the research of the structural design of the AEB control system, Bo Tang et al. [13] proposed the autonomous emergency braking pedestrian (AEB-P) system (V2V-PAEB), where vehicle-to-vehicle communication (V2V) was used to detect and identify pedestrians using the sensors and take corresponding measures. The structure and information processing of the pedestrian automatic emergency braking system (PAEB) were tested and simulated by establishing a simulation model using the MATLAB/SIMULINK. Llorca D.F. et al. [14] introduced a control system centered on pedestrian collision avoidance. This system included a stereo vision detection module that calculated the collision time between a vehicle and a pedestrian. The fuzzy controller was used to simulate human behavior and response, and the automatic vehicle collision avoidance system (CAS) was mainly based on automatic steering, which could ensure the effectiveness of pedestrian avoidance at the speed of up to 30 km/h. Song. et al. [15] put forward a new theory and algorithm to predict the position of pedestrians and determine accurate warning and braking time. This algorithm could effectively avoid or mitigate pedestrian collision accidents when the vehicle speed is below 40 km/h. Saito Y.et al. [16] designed an AEB-P system, which could control the vehicle accelerator pedal and brake pedal to guide a driver to maintain safe driving speed in a potentially dangerous driving environment wherein pedestrians who are in the blind spot of the line want to cross the road. The functionality and effectiveness of the AEB-P system were tested and verified using a driving simulator. Shimizu T. [17] developed a risk quantification model based on the collision speed in a dangerous scene where pedestrians might get out of a stopped vehicle. By comparing the consistency of this model with the real safe driving data, the effectiveness of the proposed risk assessment model in pedestrian collision risk prediction and safe driving was proved. M. A. Abu et al. [18] proposed an automotive braking system based on artificial neural networks. In this system, the brake pressure was automatically adjusted based on the distance between a vehicle and an obstacle. When the vehicle and obstacle reached the shortest safe distance, the brake pressure was maximized in order to stop the car. The simulations were carried out using the Labview simulation software platform.

S. G. Christopoulos et al. [19] proposed an emergency rear-end collision avoidance control strategy based on a hierarchical control framework consisting of a threat assessment layer and a tire slip rate control layer. In this strategy, the threat assessment layer continuously calculated the threat metrics related to collision avoidance through brake control. In the tire slip rate control layer, a radial basis function neural network (RBFNN) variable structure control (VSC) algorithm was designed to track an optimal slip rate so that controlled vehicles could produce the highest possible deceleration. The simulation test was carried out at high speed on dry and wet asphalt pavement by using MATLAB/SIMULINK platform. The results showed that the proposed AEB control scheme effectively implemented the collision avoidance strategy. I. Rizianiza et al. [20] presented an automatic braking system (ABS) design based on fuzzy logic, which was simulated on the hardware by a remote control car. The input of fuzzy logic consisted of the information about the speed and distance of an object in front of a vehicle, while the output was the braking strength.

Li et al. [21] proposed a method to detect pedestrians, cyclists, and vehicles using arrayed ultrasonic sensors and employed a conditional likelihood maximization method based on mutual information to select an optimized subset of features from the candidates. The belonging probability to each group along with time was determined based on the accumulated object type attributes outputted from a support vector machine classifier at each time step. Results showed an overall detection accuracy of 86%. The time needed for detection was about 0.8 s. Duan et al. [22] extracted the top three scenarios of V-B (vehicle-bicycle) conflicts, including SCR (a bicycle crossing from the right side while the car is running straight), SCL (a bicycle crossing from the left side while the car is running straight), and SSR (a bicycle swerving in front of the car from the right side while the car is driving straight) in China from naturalistic driving datasets. A driving simulator was employed to reconstruct these three scenarios. Results revealed that pre-decelerating behaviors were found in SCL and SSR conflicts, whereas in SCR, the subjects were less vigilant. The brake reaction time and brake severity in lateral V-B conflicts (SCR and SCL) were shorter and higher than that in longitudinal conflicts (SSR). Lian Hou et al. [23] investigated drivers’ braking behaviors in the two most typical V-B (vehicle-bicycle) conflicts with a driving simulator. The evolutionary process of the stimulus in SCR and SSR was analyzed by an eye-tracking device. The influence of motion patterns on drivers’ both prebrake and postbrake behaviors was studied through an orthogonal experiment. Results showed that drivers brake immediately when V-B conflicts occur; hence, the BRT (brake reaction time) was independent of any movement pattern parameters. They proposed a method to improve the auto-brake timing and braking phases of an adaptive bicyclist-AEB system, which could effectively solve the defects of the time to collision (TTC). Li et al. [24] presented a cost-effective approach to track moving objects around vehicles using eight linearly arrayed ultrasonic sensors and developed an empirical detection model to understand the detection characteristics of a single sensor. On the purpose of dynamic object tracking, an extended Kalman filter (EKF) and an unscented Kalman filter (UKF) were designed. The effectiveness of the designed algorithms was verified in two typical driving scenarios. Experimental results showed that both EKF and UKF had a more precise tracking position and smaller RMSE (root mean square error) than a traditional triangular positioning method. Li et al. [25] designed three experiments to examine the effectiveness of two forward crash warning systems, a flashing brake system, and a flashing hazard system. With an advanced driving simulator, experiment 1 showed that time gap, velocity, and deceleration of the lead vehicle all significantly affected drivers’ brake response times. Experiment 2 showed that the flashing brake system and flashing hazard system reduced drivers’ brake response times by 0.14∼0.62 s and 0.03∼0.95 s, respectively. Experiment 3 showed that flashing amber lamps reduced drivers’ brake response times significantly by 0.11 s (10%) on average compared with red lamps.

This paper established the architecture of the AEB-P system for the technical difficulties of the automatic emergency braking pedestrian collision avoidance system (AEB-pedestrian system, AEB-P). Using the more mature hierarchical control theory, the control structure of the AEB-P system was divided into upper layer fuzzy neural network controller and lower layer PID controller. The upper controller mainly realized the reception of the control signal and quickly and reliably outputted the desired deceleration value to the lower layer controller. Combining the longitudinal collision avoidance braking operation data of experienced pedestrians, the neural network training was carried out, and the genetic algorithm was introduced to optimize the initial parameters of the fuzzy neural network model to improve the training precision of the model. The lower controller converted the expected deceleration output of the upper controller into the throttle opening and the brake line pressure of the vehicle by establishing a vehicle inverse dynamics model to realize the actual deceleration control of the vehicle. According to the China-new car assessment program (C-NCAP) (2018 edition) issued by China Automotive Technology and Research Center, the pedestrian-vehicle test scenario was established for the pedestrian-vehicle test scenario, the CarSim and Simulink co-simulation model of the AEB-P system was established, and the results showed that the proposed control strategy was credible and reliable and could flexibly allocate early warning and braking time according to the change in actual working conditions, to reduce the occurrence of pedestrian collision accidents.

The contributions of this article are reflected in the following aspects.

(1) The AEB-P system has the functional requirements to prevent the occurrence of pedestrian collision accidents, and ensure and protect the safety of pedestrians. The architecture of the AEB-P system was proposed, and the basic functions and logical relationships of each module of the system were explained. Based on the research of collision time (TTC), the braking safety distance, and other related theoretical systems, the AEB-P system early warning model was established, which defines the driving safety level and the working area of the AEB-P warning system.
(2) Taking an E-class front-mounted SUV model as a research object, the upper-layer fuzzy neural network controller of the AEB-P system was designed. The longitudinal collision avoidance data of the experienced drivers were used as training data in the BP (backpropagation) neural network training. Based on the pedestrian avoidance test specification, relevant pedestrian test scenarios were established using Carsim software. The joint simulation model of the AEB-P system developed by using both Carsim and Simulink was designed to verify the correctness of the proposed AEB-P system control strategy.

## 2. AEB-P System Architecture

The AEB-P system mainly achieves pedestrian avoidance. Pedestrians are weak relative to a car, and pedestrian lives are precious. Therefore, the technical requirements for pedestrian avoidance of the AEB-P system are very high, and high efforts have to be made to avoid pedestrian collisions and protect their safety. Pedestrians differ in wear, behavior, gender, age, and trajectory. In the pedestrian test case, the acquired pedestrian perception information is more complicated than that of the target vehicle. Therefore, it is necessary to strictly select valid information and eliminate interference information. Pedestrian collision avoidance is mainly related to the pedestrians crossing a road. The general security algorithm cannot be fully applicable to the AEB-P system. Thus, it is necessary to integrate multiple security algorithms to ensure the effectiveness of pedestrian collision avoidance. In addition, during the emergency braking process, pedestrians should not be exposed to excessive psychological burden and injury. To this end, the functional requirements of the AEB-P system are as follows.

1. The AEB-P system should be designed such that to protect pedestrian safety as the highest priority. Under the non-extreme conditions, there should be no collision with pedestrians.
2. The AEB-P system should be more human-oriented, taking into account the psychological and physiological responses of pedestrians, and should not cause excessive panic or scare pedestrians during the emergency braking.
3. The AEB-P system should have the basic features of good functional safety, adaptability, and good robustness. In addition, the driving environment should be accurately judged, the collision risk degree should be accurately estimated, and the driver’s normal operation should not be disturbed.
4. The AEB-P system should reflect the experienced driver braking behavior and should have the ability to adaptively adjust the braking force based on changes in the danger level. It should also minimize the emergency braking process, mitigating discomfort and tension in the car occupants.
5. The AEB-P system should have self-learning and self-adaptive ability based on rich driving experience, that is, the ability to collect and store driver’s driving information and adapt to different driver’s driving habits.
6. The AEB-P system should have a collision avoidance warning function. Before the automatic intervention of emergency braking, it should be led by a driver to remind him of a potential danger of pedestrian collision.
7. The AEB-P system should meet the standards of the test regulations.

**Figure 1:**
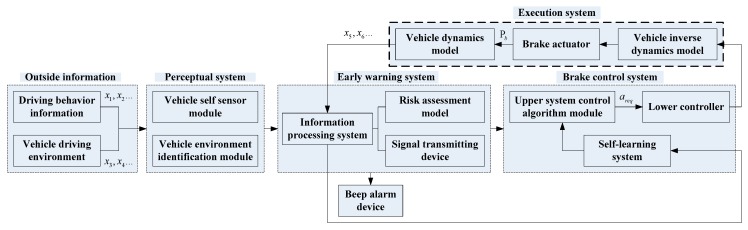
The proposed autonomous emergency braking pedestrian (AEB-P) system architecture.

Based on the above functional requirements, the AEB-P system shown in Figure 1 was designed. This system consisted of five modules, namely, sensing system, early warning system, self-learning system, control system, and execution system.

## 3. AEB-P System Model

As can be seen in Figure 1, the vehicle deceleration control requires an accurate dynamic model and an inverse dynamic model. The inverse dynamic model serves as a bridge between the AEB-P system and the vehicle dynamics model. Its main function is to convert the expected deceleration values from the AEB-P system to the parameters, such as braking force identifiable by the dynamic model. The vehicle’s brake deceleration is controlled by the established vehicle dynamic model. In the inverse dynamic model, the conversion between the expected deceleration and the vehicle braking force was studied.

### 3.1. AEB-P System Dynamic Modeling

The research object was an E-class, front-mounted front-drive SUV model. The vehicle dynamic model mainly included subsystems, such as body, aerodynamics, powertrain system, brake system, steering system, suspension, and tires. The main vehicle parameters are given in Table 1.

### 3.2. Inverse Dynamic Modeling of AEB-P System

When a vehicle begins to brake, the vehicle dynamic equation is given by:

(1) $$ma_{req}=-F_{req}-0.5C_{D}A\rho v^{2}-mgf,$$

where *F_req_* denotes the expected braking force, *C_D_* denotes the air resistance coefficient, *A* denotes the windward area, ρ denotes the air density, *m* denotes the vehicle quality, and *f* denotes the rolling friction coefficient.

When the ground-breaking force is less than the road surface adhesion, the relationship between the expected braking force *F_req_* and the brake pressure *P_req_* is given by:

(2) $$F_{req}=\frac{T_{bf}+T_{br}}{r}=K_{b}P_{req},$$

where *T_bf_* and *T_br_* are the braking torques of the front and rear wheels, respectively, *K_b_* is the ratio of the braking force to brake pressure, and the desired brake pressure is obtained by Equations (1) and (2), and it is given by:

(3) $$P_{req}=\frac{\left|-ma_{req}-0.5C_{D}A\rho v^{2}-mgf\right|}{K_{b}},$$

**Figure 2:**
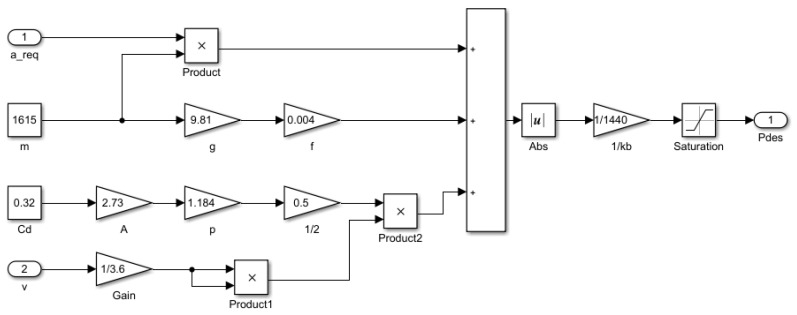
The expected braking pressure of the inverse dynamics model.

The inverse dynamics expected brake pressure model built by Simulink is presented in Figure 2.

### 3.3. Establishment of AEB-P Early Warning System

**Figure 3:**
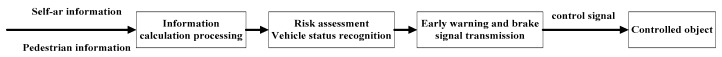
The structure of the AEB-P early warning system.

The important premise of the upper and lower controllers of the AEB-P system was to achieve accurate braking control and establish an early warning model that can accurately and timely issue early warning signals and braking signals. The AEB-P early warning system established in this study had three main modules, vehicle and driving environment information collection module, risk assessment module, and early warning and brake signal transmitting module. The structure of the AEB-P early warning system is shown in Figure 3.

As shown in Figure 3, the vehicle information included mainly information on vehicle acceleration, speed, and other vehicle moving-related parameters. On the other hand, the pedestrian information included mainly information on the relative distance, relative speed, and azimuth of pedestrians. The risk assessment module was responsible for determining the risk level based on the obtained information and security status judgment; the warning and braking signal transmitting module was responsible for issuing the corresponding control signals to the controlled object according to the risk assessment level; the controlled object was the AEB-P system. The upper controller made the corresponding control response according to the control command issued by the AEB-P warning system.

#### 3.3.1. Driving Information Acquisition and Related Calculation Processing

**Figure 4:**
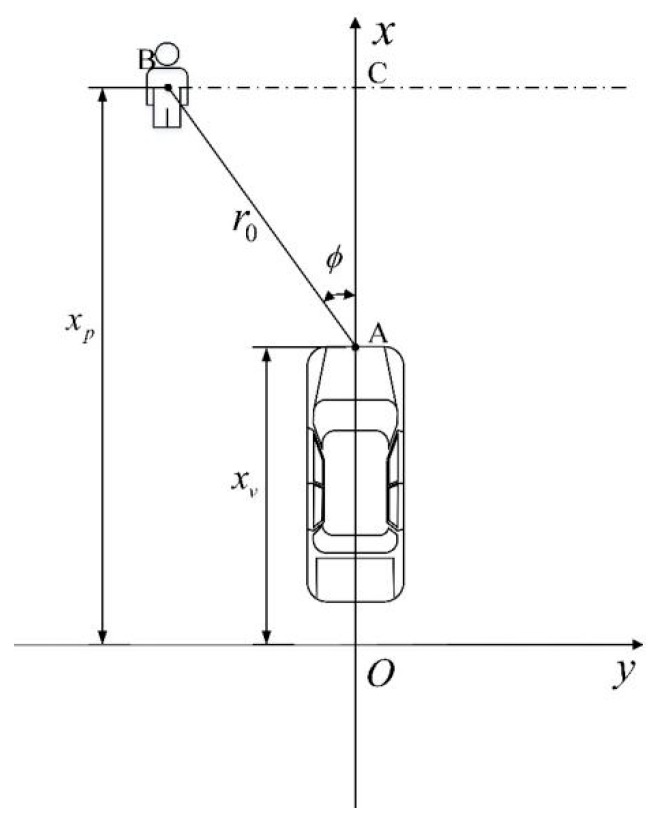
Vehicle and pedestrian position coordinates.

The vehicle sensing system was equipped with a millimeter-wave radar. The established AEB-P environment-aware coordinate system is shown in Figure 4. The self-driving direction is the positive direction of the *x*-axis, the direction of pedestrian movement near the lateral direction of a vehicle is the positive direction of the *y*-axis, the installation position of the vehicle millimeter-wave radar is point A, *x_v_* is the position of the vehicle coordinate, *x_p_* is the position of the pedestrian coordinate; the millimeter-wave radar calculates the position coordinate of the pedestrian direction vector $\vec{R_{0}}$ as ($r_{0}$, ϕ), where ϕ denotes the azimuth angle of a pedestrian relative to the travel direction of a vehicle, and $r_{0}$ is the relative position between a vehicle and a pedestrian. The lateral distance *L*_1_ of a pedestrian relative to the middle of the front end of a vehicle is given by:

(4) $$L_{1}=r_{0}\sin\phi ,$$

The relative longitudinal distance *L*_2_ between a pedestrian and the vehicle travel direction is expressed by:

(5) $$L_{1}=r_{0}\sin\phi ,$$

The obtained real-time calculation results could be used as a risk assessment model for driving safety judgment and pedestrian collision risk classification.

#### 3.3.2. Risk Assessment Model Establishment

The risk assessment model is a fundamental guarantee for the functional safety of the AEB-P system. It determines the trigger timing of the AEB-P system and has the ability to determine the driving safety status and to predict the pedestrian collision risk. As the highest decision-making layer of the AEB-P system, the risk assessment model directly affected the final result of pedestrian avoidance of the AEB-P system and thus should meet the following requirements:

1. The risk assessment model should adapt to complex pedestrian collision avoidance conditions.

The AEB pedestrian test conditional speed range was based on the simulation of urban road traffic conditions, and the pedestrian’s horizontal initial velocity, relative lateral distance, and collision location were defined accordingly.

2. Information about vehicles and pedestrians required for the risk assessment model should be readily available.

The risk assessment model should be able to analyze the acquired effective information (relative position and azimuth of pedestrians and vehicles) based on the established perception system, and then to judge the vehicle safety status and the risk of a collision.

3. The risk assessment model should be able to determine the risk level accurately and rank it.

According to different driving conditions, the risk assessment model should be able to classify the safety level reasonably so that the AEB-P system can make an early warning or braking decision-making judgments, prompting a driver to be at risk of a pedestrian collision. If the driver does not take the corresponding measures, the AEB-P system should be promptly involved and take an automatic emergency braking.

4. The risk assessment model should not interfere with the normal driving behavior of a driver.

A driver of a vehicle should not be subjected to excessive interference during the normal driving process [26]. If the warning and timing of an intervention of the risk assessment model are unreasonable, it will easily cause discomfort to the passengers in a vehicle, causing unnecessary panic and misleading the driver’s judgment and operation. Therefore, the risk assessment model is critical to the judgment of the risk degree and intervention timing.

The time to collision (TTC) is defined as the time that a driver can use to reduce the speed of a vehicle by braking to avoid collision with the front target [27]. The larger the TTC value is, the lower the risk of a collision will be, and vice versa, which is expressed as:

(6) $$t_{ttc}=\frac{-\Delta s}{v_{r}},$$

where Δs denotes the relative longitudinal distance between a vehicle and a target. However, Equation (6) [27] does not hold when $v_{r}=0$. $v_{r}=0$ when the relative distance between a vehicle and a target is too close, therefore, the relative acceleration needs to be introduced to meet the needs of more complicated working conditions. After introducing the relative acceleration [28] $a_{r}$, we got:

(7) $$\Delta s+v_{r}t_{TTC}+\frac{1}{2}a_{r}t_{TTC}^{2}=0,$$

Equation (6) can be further expressed as:

(8) $$t_{TTC}=\left\{ \begin{array}{ll} -\frac{\Delta s}{v_{r}} & v_{r}<0\;\&\;a_{r}=0\\ -\frac{v_{r}}{a_{r}}-\frac{\sqrt{{v_{r}}^{2}-2\Delta sa_{r}}}{a_{r}} & v_{r}<0\;\&\;a_{r}\neq 0\\ -\frac{v_{r}}{a_{r}}+\frac{\sqrt{{v_{r}}^{2}-2\Delta sa_{r}}}{a_{r}} & v_{r}\geq 0\;\&\;a_{r}<0 \end{array}\right.,$$

However, after the introduction of $a_{r}$, there are still cases where Equation (8) is not applicable. When $v_{r}\geq 0\;\&\;a\geq 0$ and ${v_{r}}^{2}-2\Delta sa_{r}<0$, Equation (8) has no solution. At the same time, after a vehicle starts to brake, the speed, relative distance, and deceleration are constantly changing. Thus, the TTC risk assessment is likely to be misjudged, resulting in false alarms or failed alarms. Therefore, further research on the vehicle braking process is needed to enhance the reliability of the risk assessment model.

#### 3.3.3. Classification of Pedestrian Collision Hazard Level and Analysis of Braking Process

**Figure 5:**
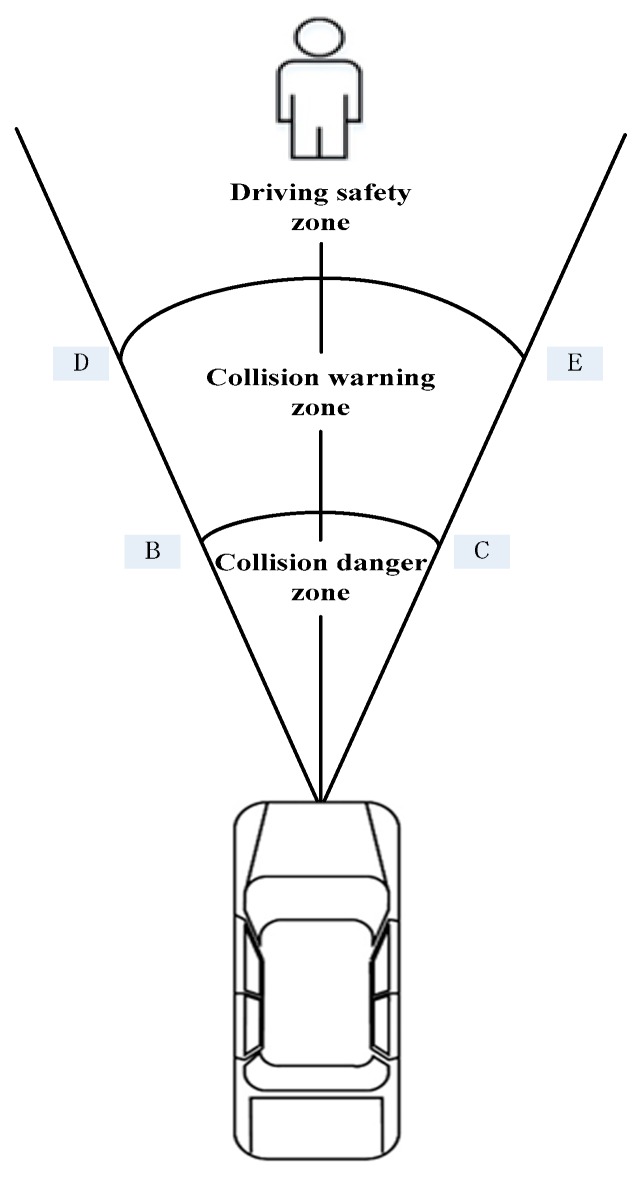
Security classification region of the AEB-P system.

The established AEB-P early warning system uses a hierarchical early warning algorithm to classify the security levels at different driving states. Different security levels correspond to different TTC values. According to the degree of danger, the security levels are divided into three levels; the first level is the driving safety level, the second level is the collision warning level, and the third level is the collision danger level. The first level indicates the current driving safety without potential danger, and in that case, the AEB-P warning system sends a signal value of 0. The second level indicates that the pedestrian has been detected, and there is a potential collision risk, so a driver needs to take the corresponding braking measures, and the AEB-P warning system sends a signal value of one. The third level denotes an impending pedestrian collision, and the AEB-P warning system sends a brake signal value of two. If a driver does not take the necessary braking measures, the brake system will automatically intervene to avoid the collision. The AEB-P system does not intervene when a vehicle is in a safe driving class. When the vehicle is in the collision warning level, the system will continue to sound an alarm tone. The AEB-P system regional security level is divided, as shown in Figure 5.

**Figure 6:**
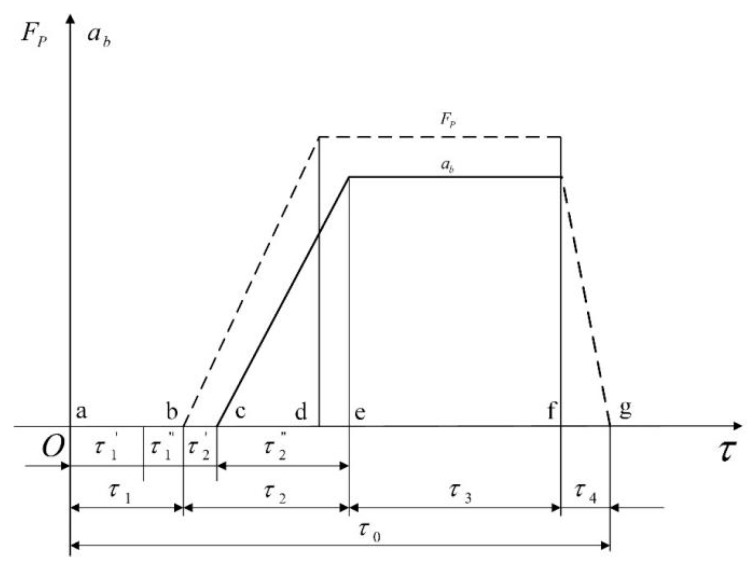
Vehicle braking process.

After dividing the AEB-P system into three hierarchical warning levels, it is necessary to divide also the TTC time interval into the corresponding levels. First of all, the warning time of the second level (warning level) should be determined. The alarm time of the second level warning is related to the credibility of the AEB-P warning system and the final collision avoidance result. If the alarm time is too short, a driver has no time to take relevant measures, causing the system not to play the role of pedestrian collision warning. On the contrary, if the alarm time is too long, the warning will interfere with the normal operation of a driver, resulting in a driver’s feelings of boredom and distrust of the AEB-P warning system. Thus, the alarm duration of the second-level warning is an important parameter of the AEB-P warning system. Therefore, the driver response time, the elimination of the brake gap time, and the hydraulic lag time should be fully considered. Moreover, to determine a reasonable warning time, the vehicle braking process should be accurately analyzed. The vehicle braking process is shown in Figure 6.

In analyzing the vehicle braking process, the following parameters are used: $\tau_{1}^{\prime }$ denotes the time interval during which a driver receives the warning information and realizes the braking process, $\tau_{1}^{\prime \prime }$ denotes the time for a driver to perform the braking action, and the reaction time of a driver which is expressed as $\tau_{1}=\tau_{1}^{\prime }+\tau_{1}^{\prime \prime }$. Recent studies have shown that the driver’s response time is generally from 0.3 s to 1 s. After point b, the braking force $F_{p}$ and the braking deceleration of a vehicle increase rapidly, reaching the maximum at points d and e, respectively. The brake-clearance time is $\tau_{2}^{\prime }$, the brake-power-increasing time is $\tau_{2}^{\prime \prime }$, and the brake-action time $\tau_{2}=\tau_{2}^{\prime }+\tau_{2}^{\prime \prime }$, $\tau_{2}$ is generally between 0.2 s and 0.9 s.

**Figure 7:**
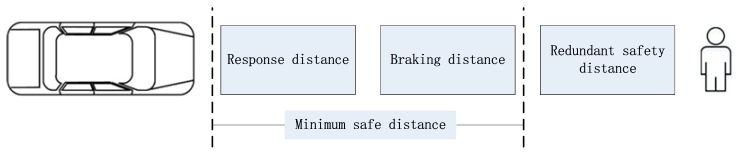
Vehicle braking safety distance.

The warning time of the AEB-P early warning system should fully consider the driver’s reaction time and brake action time [29]. The complexity and danger-level of pedestrian test conditions were considered in the study. The warning time of the AEB-P system was set to 1.5 s. In order to avoid the failure condition of the TTC theoretical model when determining the TTC interval of the third level collision hazard, the vehicle braking safety distance was also analyzed. As shown in Figure 7, the braking safety distance of a vehicle consists of three parts: response distance, braking distance, and redundant safety distance. The sum of the response distance and braking distance is called the minimum safety distance. The redundant safety distance means that after the vehicle completes the emergency braking, it should keep a certain distance from a pedestrian, to avoid making a close distance between the vehicle and pedestrians and causing unnecessary panic to pedestrians.

The response distance is the braking distance at which the brake starts to act, and the brake-deceleration increases linearly. The braking distance is a distance that the vehicle has traveled from the moment it starts to apply the brake to the moment when it stops, i.e., its speed is zero. The vehicle brake safety distance is given by:

(9) $$d=\frac{u_{0}}{3.6}(\tau_{2}^{\prime }+\frac{1}{2}\tau_{2}^{\prime \prime })+\frac{u_{0}^{2}}{25.92a_{bmax}}+d_{0},$$

where $a_{b\max}$ denotes the vehicle stabilized deceleration, $u_{0}$ denotes the initial vehicle speed, and the redundant safety distance $d_{0}$ is about 2 m. According to Equation (9), the vehicle brake safety distance is related to the brake action time, the initial vehicle speed, and the maximal vehicle deceleration. The maximum acceleration of a vehicle can generally reach 10 m/s^2^, and the greater the initial vehicle speed is, the longer the safer braking distance is. Therefore, the integrated safety distance theory and the established risk assessment model can be expressed as:

(10) $$t_{oTTC}=\left\{ \begin{array}{ll} -\left(\frac{1}{3.6}(\tau_{2}^{\prime }+u_{0}\tau_{2}^{\prime \prime })+\frac{u_{0}}{25.92a_{bmax}}+\frac{2}{u_{0}}\right) & v_{r}<0\;\&\;a_{r}=0\\ -\frac{v_{r}}{a_{r}}-\frac{\sqrt{{v_{r}}^{2}-2a_{r}(\frac{u_{0}}{3.6}(\tau_{2}^{\prime }+u_{0}\tau_{2}^{\prime \prime })+\frac{u_{0}^{2}}{25.92a_{bmax}}+2)}}{a_{r}} & v_{r}<0\;\&\;a_{r}\neq 0\\ -\frac{v_{r}}{a_{r}}+\frac{\sqrt{{v_{r}}^{2}-2a_{r}(\frac{u_{0}}{3.6}(\tau_{2}^{\prime }+u_{0}\tau_{2}^{\prime \prime })+\frac{u_{0}^{2}}{25.92a_{bmax}}+2)}}{a_{r}} & v_{r}\geq 0\;\&\;a_{r}<0 \end{array}\right.,$$

where $t_{0TTC}$ denotes the brake safety threshold. When $TTC\leq t_{0TTC}$, the risk assessment model will issue a brake control signal; when $t_{0TTC}\leq TTC\leq t_{0TTC}+1.5$, the risk assessment model will issue an early warning control signal; lastly, when $TTC\geq t_{0TTC}+1.5$, representing the driving safety, the risk assessment model will not issue a control signal.

This study, relying on the empirical driver TTC statistics [30] and following the vehicle braking safety distance theory, determined the TTC values range at different safety levels. The test conditions were divided based on the initial vehicle speed, the TTC value range, and the corresponding safety level, as shown in Table 2. The TTC values range of the third-level collision danger zone is related to the initial vehicle velocity. The larger the initial velocity is, the larger the TTC value range of the third-level collision danger zone is. For the TTC value range that satisfies the second-level collision warning level, the AEB-P system will continuously emit a buzzer alarm. When the TTC value range corresponds to the first level, the vehicle has no potential collision risk, and the AEB-P system will not interfere with the driver’s normal driving.

In addition, when the vehicle enters the third level, due to frequent changes in vehicle deceleration, the computational burden of the risk assessment model will increase, and false alarms are likely to occur. Therefore, after entering the third level, the warning system will continue to issue the brake signal, and the TTC value will not be calculated.

### 3.4. Division of AEB-P System Operating Area

**Figure 8:**
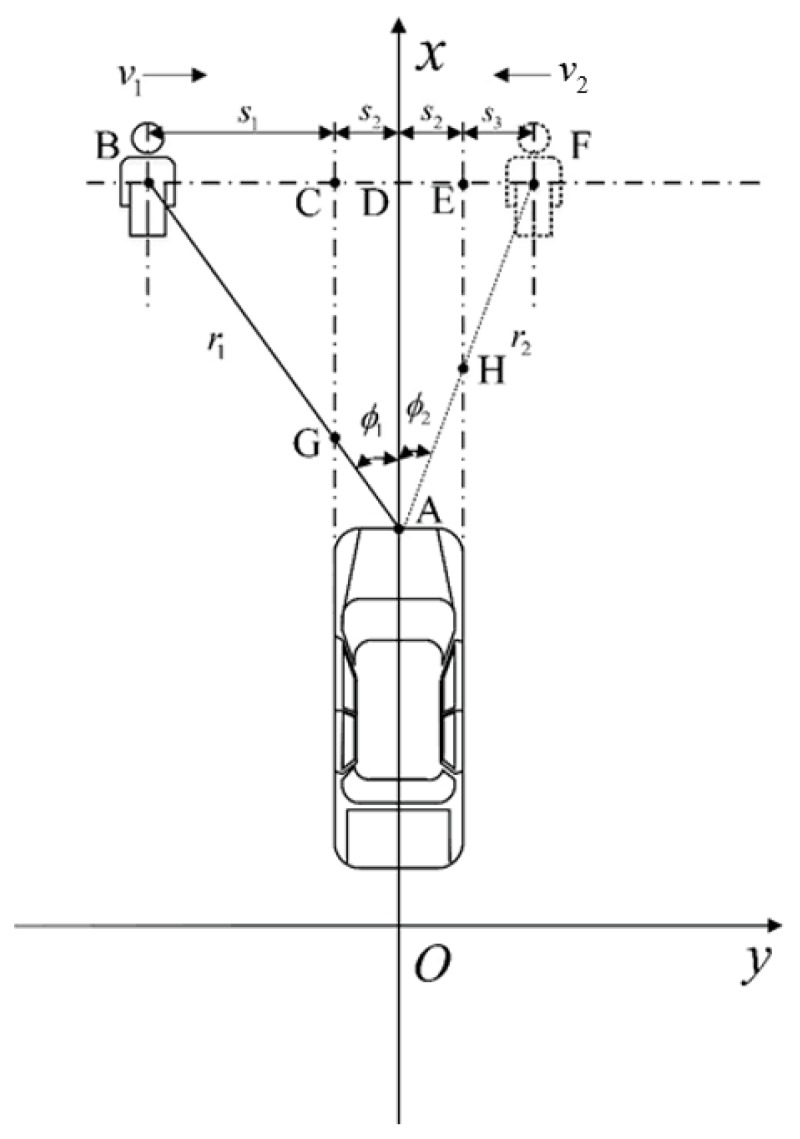
Working area division of the AEB-P system.

As shown in Figure 8, when the AEB-P risk assessment model is used under complex traffic conditions, there is a problem. The working area of the AEB-P system is divided to ensure that the AEB-P warning system is accurately activated. When a self-car is close enough to a pedestrian (the TTC division in Table 2 is used as a standard), the AEB-P system may be in off, ready to work, or active state, which is mainly based on the pedestrian status as a reference indicator. If the pedestrian is in the BCG or EFH area (refer to Figure 8), and $v_{1}\neq 0$ or $v_{2}\neq 0$, the AEB-P system will be in a ready working state. In this case, the risk assessment model will not issue any control signals, and the system will prompt a driver to pay attention to pedestrian safety in the form of an image. If a pedestrian is in the AECGH area, the AEB-P system will be activated regardless of the sporting state of a pedestrian. In this case, the risk assessment model will issue a pedestrian collision warning or an automatic emergency braking signal. Combined with the C-NCAP test condition standard [10], we defined $s_{1}=5.05\;m$ and $s_{3}=3.05\;m$, and according to the E-class SUV parameters, we obtained $s_{2}=0.95\;m$.

## 4. AEB-P System Controller Design

### 4.1. AEB-P System Upper Controller Design

The key to achieving pedestrian vertical active collision avoidance in the AEB-P system is to control the vehicle dynamics. The upper controller acts as a control core of the entire AEB-P system. Its main function is to receive control signals and to output the desired deceleration value to the lower controller quickly and reliably. In the braking control process, it is necessary to consider the safety of both pedestrians and drivers, but also to take into account the comfort during braking and avoid the braking process interference with the driver’s normal driving, which reflects its multi-targeting during the emergency collision avoidance, the judgment of a potential danger, the timing of braking, the magnitude of braking force, etc., which all depend on the driver subjective feelings and his experience; this reflects the ambiguity of the AEB-P system. Therefore, the fuzzy neural network model was used to design the upper decision controller to meet the basic requirements of the vehicle brake control system.

#### 4.1.1. Establishment of AEB-P Fuzzy Control System

① Determination of input and output parameters of the fuzzy control system

According to reference [11], the input of the fuzzy control system consists of the relative longitudinal speed $v_{r}$ ($\Delta v_{0}=0-v_{2}$, given in km/h) and the relative longitudinal distance Δs (m) of a vehicle and a pedestrian, where the longitudinal speed of a pedestrian is 0, and the longitudinal speed of the vehicle is $v_{2}$, while the fuzzy control system output is expected acceleration $a_{r}$ (m/s^2^). The range of the relative longitudinal velocity $v_{r}$ fuzzy domain is (−80, 0), the longitudinal relative distance Δs fuzzy domain is in the range (0, 50), and the fuzzy control system output $a_{r}$ is in the range (−10, 0).

**Figure 9:**
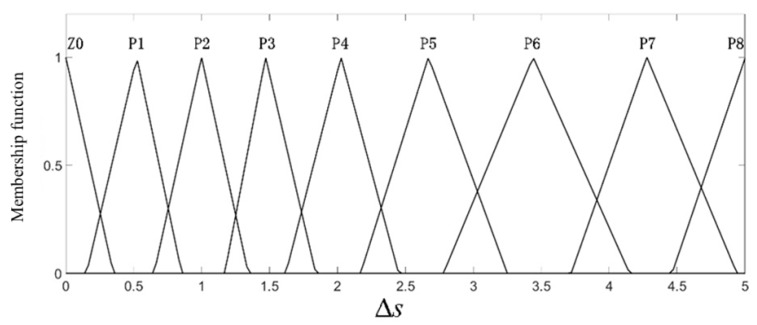
The membership function of the longitudinal relative distance.

**Figure 10:**
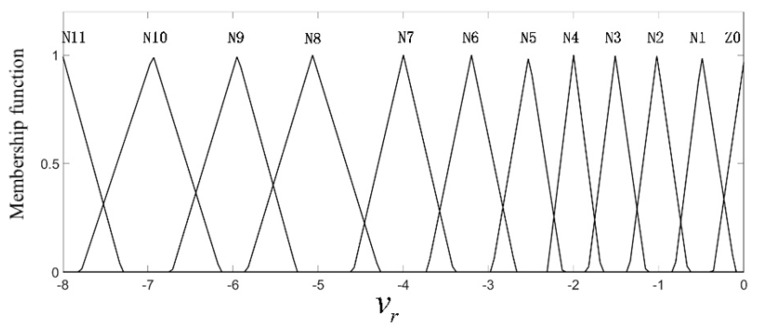
The membership function of the longitudinal relative velocity.

**Figure 11:**
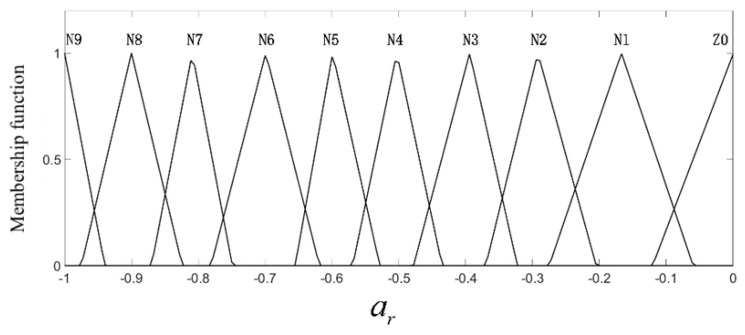
The membership function of the expected deceleration.

To improve the convergence speed of the BP neural network using the gradient descent method for the model training and to improve the training accuracy, the input and output data need to be normalized. If the actual values are in the range (a, b), and should be normalized to the range (c, d), then the normalization equations are as follows:

(11) $$x_{r}=\frac{c+d}{2}+k_{b}\left(x-\frac{a+b}{2}\right),$$

(12) $$k_{b}=\frac{d-c}{b-a},$$

where $k_{b}$ is the scale factor, and here, it was equal to 0.1. In this work, the range of the relative longitudinal speed after normalization was (−8, 0), the normalized range of the relative longitudinal distance was (0, 5), and the output value, i.e., $a_{r}$, was in the range (−1, 0).The fuzzy controller input and output linguistic variables were divided into several fuzzy sets, namely N11 (negative large)–N1 (negative small), Z0 (zero), P1 (positive small)–P8 (positive large), and the triangular membership function was used to achieve fuzzy neural network adaptation and self-learning requirements. The input and output membership functions are shown in Figure 9, Figure 10 and Figure 11.

In Figure 9, the relative vertical distance Δs is described by nine triangular membership functions, namely Z0–P8; when it is small, the membership function is more densely distributed, which means that the collision risk is higher. In Figure 10, the longitudinal relative velocity $v_{r}$ has a total of 12 triangular membership functions, namely Z0–N11; the smaller the value of $v_{r}$ is, the closer the car to the pedestrians and the higher the collision risk is. Therefore, there are seven membership functions distributed in the domain (−3, 0). In Figure 11, the expected deceleration $a_{r}$ has a total of 10 membership functions; when the value of $a_{r}$ is large, the membership function is densely distributed. According to the distribution of the membership function of each input and output quantity, the central value and width of the membership function of N11–N1, Z0, P1–P8 can be obtained initially, and they are shown in Table 3, Table 4 and Table 5.

② Establishment of fuzzy rules of the fuzzy control system

The fuzzy rules simulate the logical judgment process of a driver in the case of a potential pedestrian collision hazard. As mentioned, the established fuzzy control system has two inputs (Δs, $v_{r}$) and one output $a_{r}$. The basic control rule should satisfy the following: when Δs is small, and $v_{r}$ is large, then $a_{r}$ is large; when Δs is large, and $v_{r}$ is small, then $a_{r}$ is smaller. The formulated fuzzy control rules are given in Table 6.

According to the Mamdani fuzzy inference model, the AEB-P system fuzzy rules can be expressed as follows:

If Δs is Z0 and $v_{r}$ is Z0, $a_{r}$ is N1;
If Δs is Z0 and $v_{r}$ is N1, $a_{r}$ is N5;
If Δs is Z0 and $v_{r}$ is N2, $a_{r}$ is N7;
…
If Δs is Z0 and $v_{r}$ is N11, $a_{r}$ is N9;
If Δs is P1 and $v_{r}$ is Z0, $a_{r}$ is Z0;
If Δs is P1 and $v_{r}$ is N1, $a_{r}$ is N2;
…
If Δs is P8 and $v_{r}$ is N9, $a_{r}$ is N6;
If Δs is P8 and $v_{r}$ is N10, $a_{r}$ is N7;
If Δs is P8 and $v_{r}$ is N11, $a_{r}$ is N8;
…
If Δs is P8 and $v_{r}$ is N9, $a_{r}$ is N6;
If Δs is P8 and $v_{r}$ is N10, $a_{r}$ is N7;
If Δs is P8 and $v_{r}$ is N11, $a_{r}$ is N8.

There are a total of 108 rules. The basis of each rule usage is the degree of matching with the state of the current input parameter. The higher the matching degree is, the more the rule is used, and the corresponding output parameter is inferred.

#### 4.1.2. Training of Fuzzy Neural Network Model

**Figure 12:**
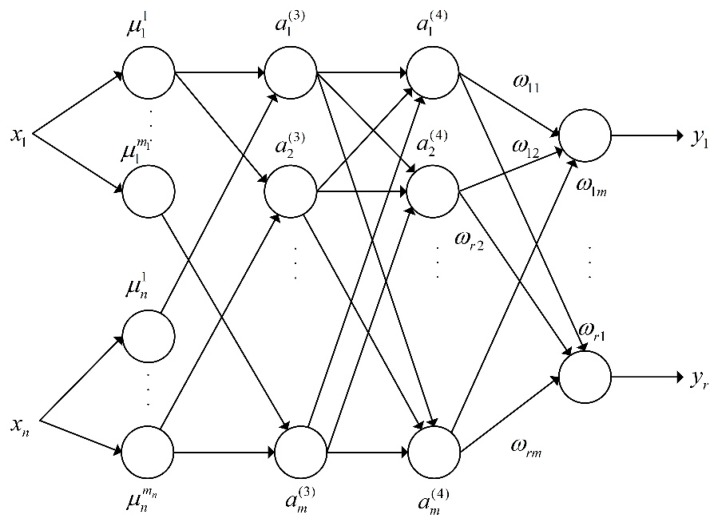
The structure of the fuzzy neural network.

Artificial neural networks can be divided into two basic types: feedforward neural networks and feedback neural networks. The neuron of the feedforward neural network receives only the output of the previous layer as its input and passes its output to the next layer. There is no feedback loop in the whole process. The feedback neural network has at least one feedback loop that can achieve reverse propagation of the signal. The feedforward neural network is a learning network with strong classification and pattern recognition capabilities and can handle complex nonlinear problems. The typical feedforward neural networks include multilayer perceptrons (MLP), error backpropagation networks (BP), etc. The feedback neural network requires a certain period to stabilize the output due to the existence of a feedback loop. The learning ability and decision-making ability of the neural network are limited. The fuzzy inference model is generally divided into a Mamdani fuzzy inference model and Tagagi–Sugeno (T-S) fuzzy inference model. The T-S fuzzy inference model is a linear equation, which is suitable for studying simple linear systems. The Mamdani fuzzy inference model can meet the research requirements of complex nonlinear systems with good reliability. Therefore, this paper choses the BP feedforward fuzzy neural network based on a Mamdani fuzzy inference model, and its network structure is shown in Figure 12.

In Figure 12, the fuzzy neural network has five layers, one input and one output, and three hidden layers. The *x* is the first input layer, and the number of nodes directly connected to the respective components of the input vector $x={\left[x_{1}x_{2}\ldots x_{n}\right]}^{T}$ is $N_{1}=n$. Each node in the second layer represents a linguistic variable for calculating the membership function of each input component and characterizing the degree of membership of each linguistic variable. The *n* is the dimension of the input *x* dimension, *m_i_* is the number of divisions of *x_i_*, and the total number of nodes is $N_{2}=\sum_{i=1}^{n}m_{i}$. The third layer is the node rule layer, and $a_{p}^{\left(3\right)}$ represents the applicability of each rule. The fourth layer is the normalized layer, and the number of nodes is the same as the third layer, that is $N_{4}=m$. The fifth layer is the output layer, which converts the amount of blur into a clear value output. The fuzzy neural network model of the AEB-P system is trained using the aforementioned fuzzy control system input and output parameters. A total of 187 training samples were selected in this paper, and some of them are given in Table 7.

The experienced driver’s data on the brake working condition form a test vehicle in a vehicle test site in southwestern China. The professional vehicle driver controls the loading data acquisition and signals processing equipment according to the pedestrian test conditions. Braking test was performed during the daytime with good weather conditions and sufficient light, and data on the braking process were collected.

In view of the fact that there is no uniform standard for the division of professional and non-professional drivers at home and abroad, this paper defined a driver who is driving for more than 7 years, has a cumulative trip of more than 100,000 km, and has accumulated more than 40,000 km in the past 2 years as a professional driver.

This experiment mainly collected the relative longitudinal speed, relative distance, and self-vehicle acceleration information. The data acquisition equipment selected 77GHZ FR-51F millimeter-wave radar. The radar was installed at the center of the vehicle intake grille, and the sampling frequency was 20 times/s. After the experiment was completed, the obtained raw data was processed, including eliminating missing or erroneous data and calculating driver behavior characteristics indicators (relative longitudinal distance, relative speed, self-vehicle acceleration, self-vehicle speed, etc.) of the pedestrian braking condition.

① Determination of learning rate of the fuzzy neural network model

In the process of fuzzy neural network training, the magnitude of the learning parameter update was determined by the learning rate. The exponential decay method was employed to update the learning rate in each training epoch. Since the input parameters and the connection weights of the membership function are respectively in different structural layers of the fuzzy neural network, the initial learning rate and the attenuation rate should also be different; otherwise, the training efficiency and training results may be reduced. In this study, the learning rate of the second layer and the last layer $\beta_{1}$ and $\beta_{2}$ were set to:

(13) $$\beta_{1}=0.2\cdot 0.95^{\frac{n}{16}},$$

(14) $$\beta_{2}=0.005\cdot 0.95^{\frac{n}{18}},$$

② Fuzzy neural network model initial parameters setting and training flowchart

**Figure 13:**
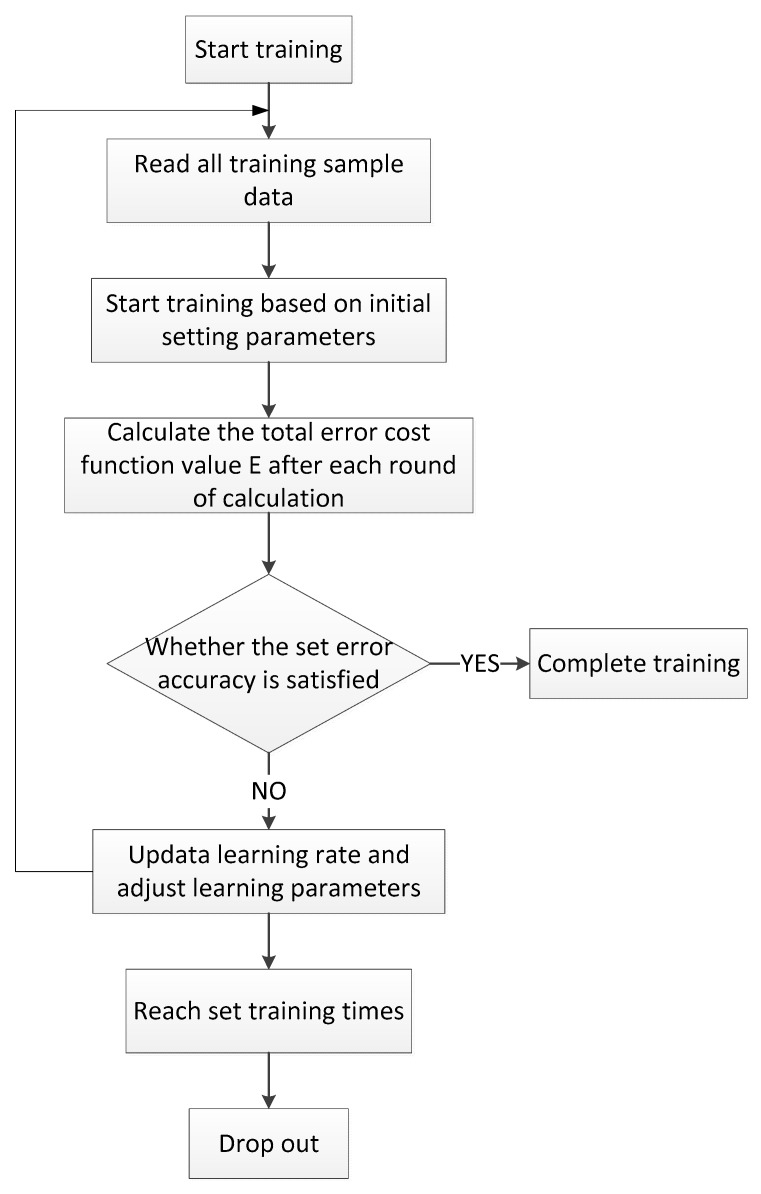
The workflow of the fuzzy neural network.

The established fuzzy neural network model adopted the error backpropagation learning algorithm. The initial values of the learning parameters ω, c, $\sigma^{\prime }$, and $\sigma^{\prime \prime }$ were randomly generated from the range (−1, 1). The total number of training epochs was set to 800, and the error cost function was determined. The code was written in MATLAB. The specific fuzzy neural network training flowchart is shown in Figure 13.

③ Fuzzy neural network model training results

**Figure 14:**
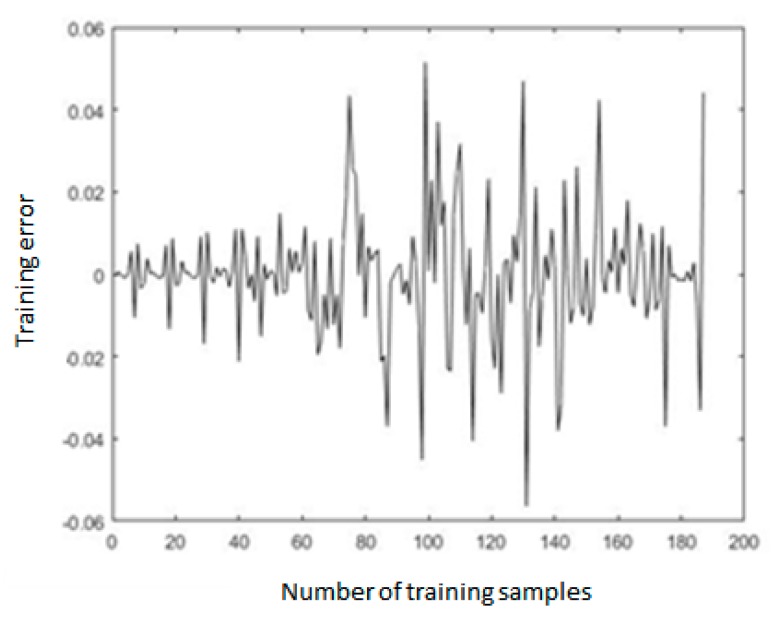
The distribution of sample error.

**Figure 15:**
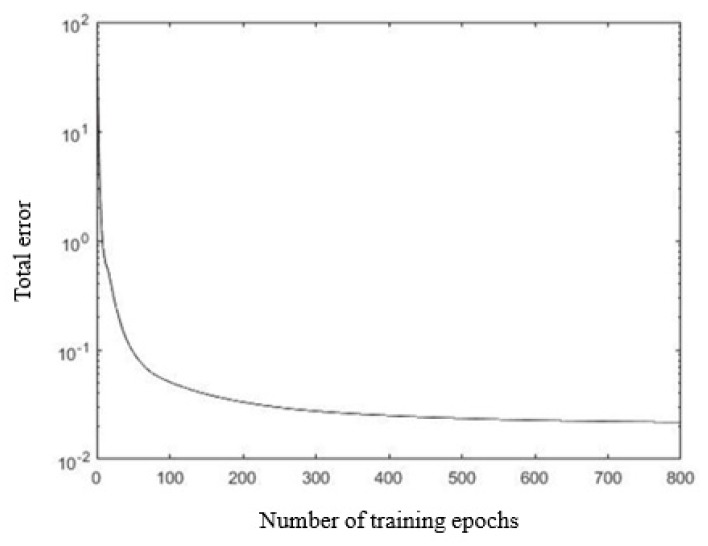
The variation trend of the total error.

**Figure 16:**
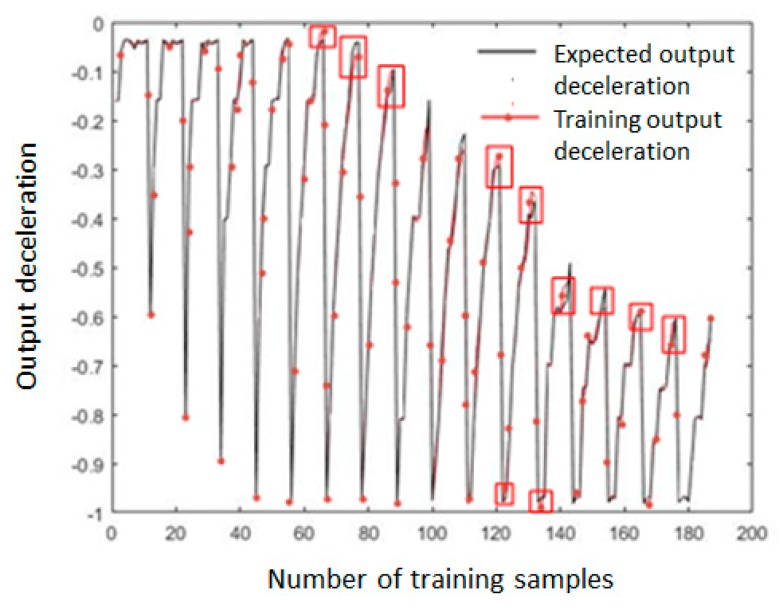
The comparison of expected and training deceleration values.

The MATLAB was used for model training, and the total training time was 62.16 s. The error distribution, the total error trend, and the expected deceleration value and training output deceleration value of 187 sets of data samples are shown in Figure 14, Figure 15 and Figure 16. As shown in Figure 14, the error of all the training samples was controlled between −0.04 and 0.04, the error of 12 datasets was between 0.04 and 0.06, and the maximum error was −0.058. As shown in Figure 15, after the training starts, the total error *E* of the sample rapidly decreased below 0.1. After the number of training epochs reached 100, the error decreasing trend became slower. After 800 training epochs, the total error *E* was 0.043.

**Figure 17:**
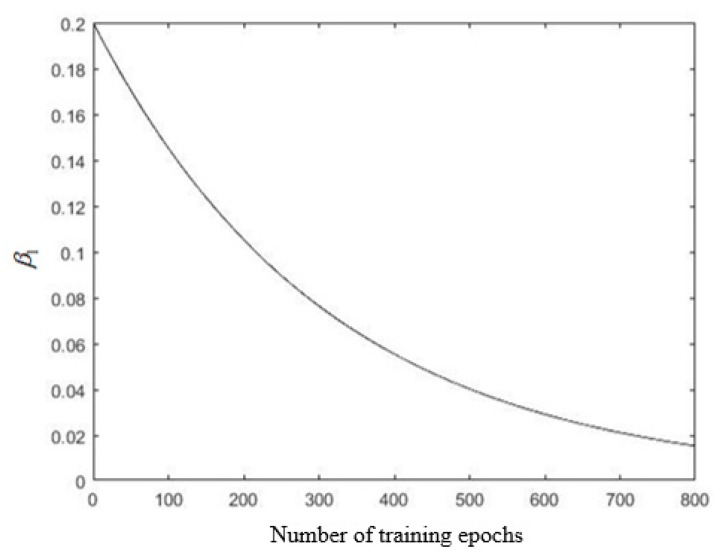
The variation trend of learning rate $\beta_{1}$.

**Figure 18:**
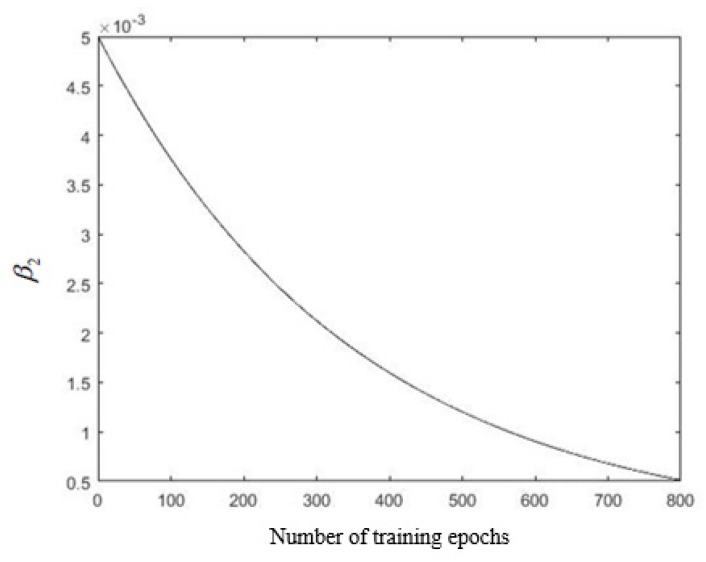
The variation trend of learning rate $\beta_{2}$.

In Figure 16, the expected deceleration denotes the actual sample output parameter value, and the training output deceleration is the output parameter value obtained by the fuzzy neural network after training and parameter optimization; it can be seen that the degree of coincidence with the expected deceleration was good, and a more obvious error occurred only in the red frames marked in Figure 16. Thus, it was shown that the error BP algorithm combined with a stepwise optimization was effective in controlling the error. Changed learning rates $\beta_{1}$ and $\beta_{2}$ are shown in Figure 17 and Figure 18, respectively. The initial value of $\beta_{1}$ was set to a large value of 0.2, and it was exponentially decaying during the training, and the final value was 0.0154.

The initial value of $\beta_{2}$ was set to a small value of $5\times 10^{-3}$, and the value after the training ended was $5.116\times 10^{-4}$.

### 4.2. AEB-P System Lower Controller Design

**Figure 19:**
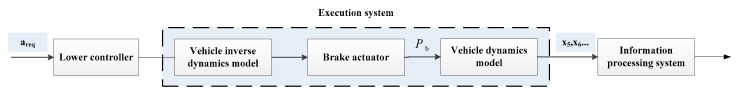
The structure of the lower controller.

The function of the lower controller is to control the dynamic system of a vehicle using the established inverse dynamic model and convert the expected deceleration output of the upper controller into the throttle opening and brake line pressure of a vehicle to realize the vehicle actual deceleration control. The structure of the lower controller is shown in Figure 19.

In Figure 19, *P_b_* denotes the basic driving information, such as speed and displacement, during vehicle operation. The design of the underlying controller should be focused on the controller robustness and stability, as well as its ability to correct and feedback errors.

Taking the AEB-P system as a research object, the lower layer PID control law is expressed as [31]:

(15) $$u(t)=K_{P}\left[e(t)+\frac{1}{T_{I}}\int_{0}^{t}e(t)dt+\frac{T_{D}de(t)}{dt}\right],$$

where $T_{I}$ is the integral time constant, $T_{D}$ is the differential time constant, and the relationship between $K_{I}$, $K_{D}$ and $K_{P}$, $T_{I}$, and $T_{D}$ is given by:

(16) $$K_{I}=\frac{K_{P}}{T_{I}},K_{D}=K_{P}T_{D},$$

To fully guarantee the control accuracy of the AEB-P system lower controller, the test conditions of the AEB-P system were divided based on the initial vehicle speed. The PID parameter tuning method is generally divided into two types: one is the theoretical calculation tuning method, which requires an accurate system mathematical model and transfer function, and three parameters are determined through theoretical calculation, and then need to be adjusted and modified through engineering practice. The second is the engineering trial and error method, which mainly relies on engineering experience. Through a large number of tests, the parameters are adjusted according to the engineering experience formula.

Because the AEB-P system research object is a complex, multi-target vehicle brake control nonlinear system, coupled with the complex variability of the driving environment, it is difficult to establish an accurate mathematical model to solve the three parameters of PID control. Therefore, this paper used the empirical trial and error method to set the PID parameters.

**Figure 20:**
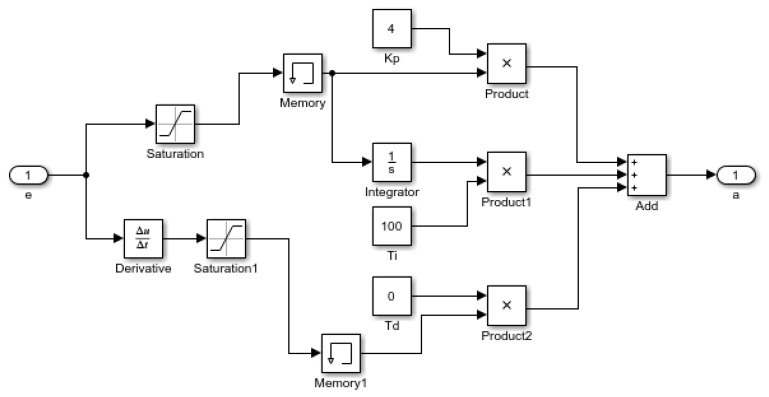
The PID lower controller model.

After a large number of tests and repeated verification, the PID values under different working conditions were as shown in Table 8. The structure of the lower layer PID controller model is shown in Figure 20.

## 5. AEB-P System Joint Simulation Analysis

### 5.1. AEB-P System Pedestrian Collision Avoidance Test Conditions

**Figure 21:**
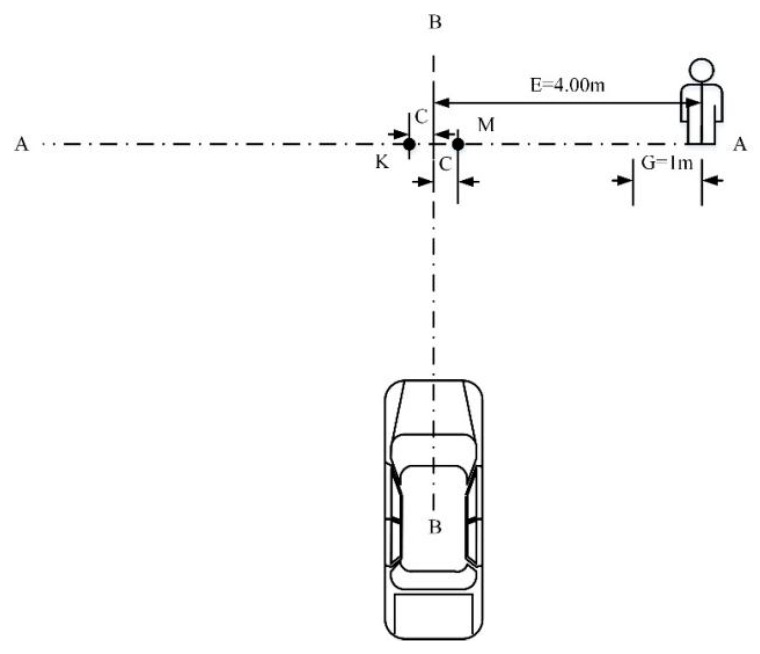
The CVNA (Car-to-VRU Nearside Adult)-25 andCVNA-75 (near-end).

**Figure 22:**
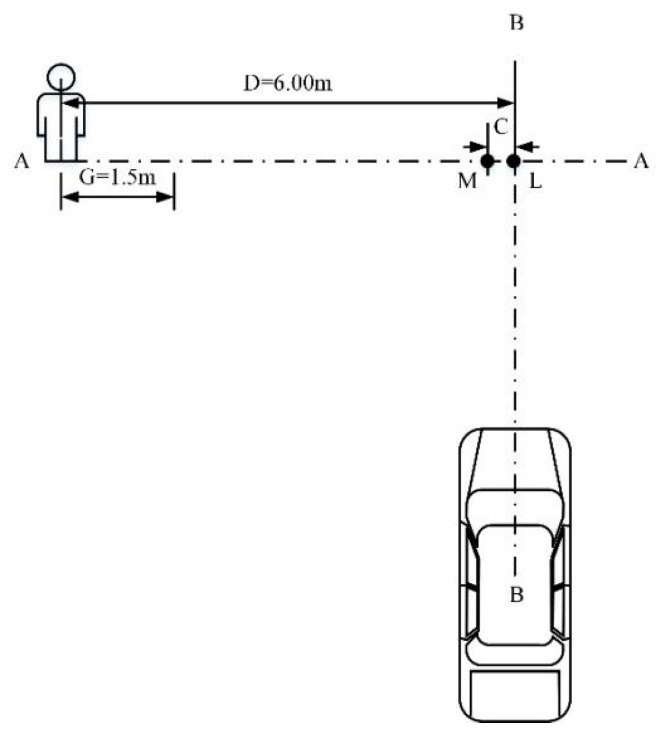
The CVFA (Car-to-VRU Farside Adult)-50 andCVFA-25 scenarios (far-end).

Based on reference [10], a total of four test scenarios of the C-NCAP pedestrian automatic emergency braking system test were designed, namely, CVFA (Car-to-VRU Farside Adult)-25, CVFA-50, CVNA (Car-to-VRU Nearside Adult)-25, and CVNA-75. According to the different pedestrian positions, the scenarios were divided into pedestrian near-end (CVNA) and pedestrian far-end (CVFA). The CVNA and CVFA test scenarios were then divided according to the position where the vehicle front and pedestrian collided into 25% collision (CVFA-25, CVNA-25), 50% collision (CVFA-50), and 75% collision (CVNA-75) scenarios. In the test scenarios, the pedestrian walking speed was 5 km/h and 6.5 km/h. The pedestrian collision avoidance test conditions of the AEB-P system are shown in Figure 21 and Figure 22.

**Figure 23:**
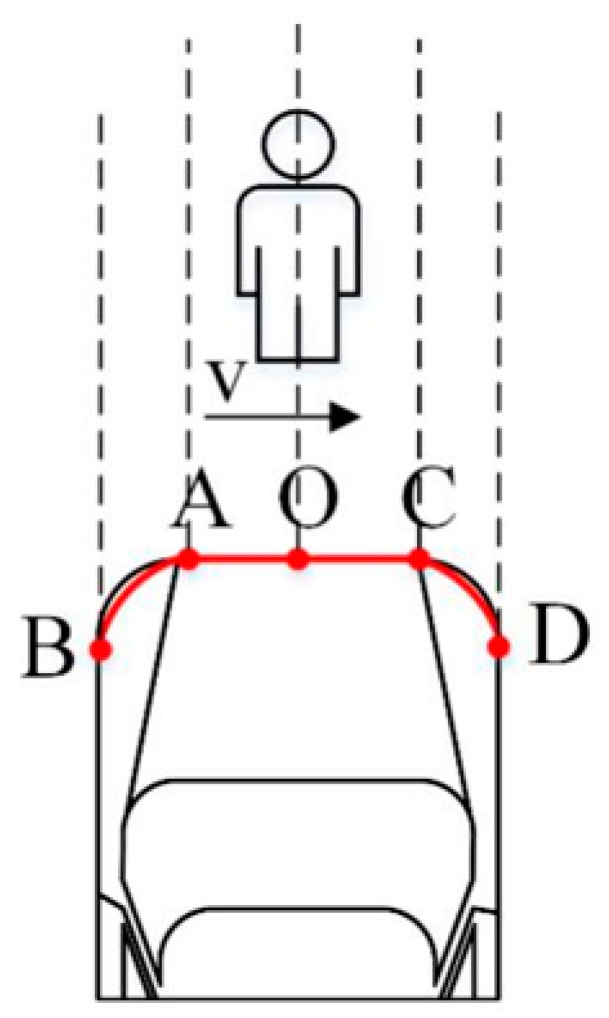
Pedestrian collision location.

In Figure 21, the axis AA denotes the motion trajectory of the dummy center point H, BB is the test vehicle centerline, G is the dummy acceleration distance, and C denotes the 25% or 75% near-end scene collision position offset; K is the 75% collision position of the near-end scene, and M is the 25% collision position of the near-end scene. In Figure 22, C is the 25% far-end scene collision position offset, L is the 50% far-end scene collision position, and M is the 25% far-end scene collision position. The position where a specific pedestrian collided with the front of the vehicle is shown in Figure 23. When pedestrians crossed the road to the right, point A was a 25% collision position, point O was 50% collision position of vehicle head center, and point C was a 75% collision position.

The test conditions were divided into five groups according to the vehicle speed. The speed was in the range 20–60 km/h, and the speed step was 10 km/h. The test scenarios corresponding to the test condition were CVFA-50, CVFA-25, CVNA-25, and CVNA-75. The pedestrian speed in the CVFA scene was 6.5 km/h, and the pedestrian speed in the CVNA scene was 5 km/h. The test scenario of the AEB-P system is given in Table 9.

In the test, there were requirements for dry pavement, no visible moisture on the surface, flatness, singleness, firmness, single slope, maintained from 0% to 1%, and peak adhesion coefficient greater than 0.9. A three-dimensional pedestrian model was made based on the appearance of an average adult male Chinese. Under the premise of meeting the test requirements, the dummy model was used as a test target of the AEB-P system to simulate the pedestrian crossing the road in the vehicle emergency collision avoidance test. The scene and the pedestrian avoidance scenario test database were built.

### 5.2. AEB-P System Pedestrian Test Scenario Construction

**Figure 24:**
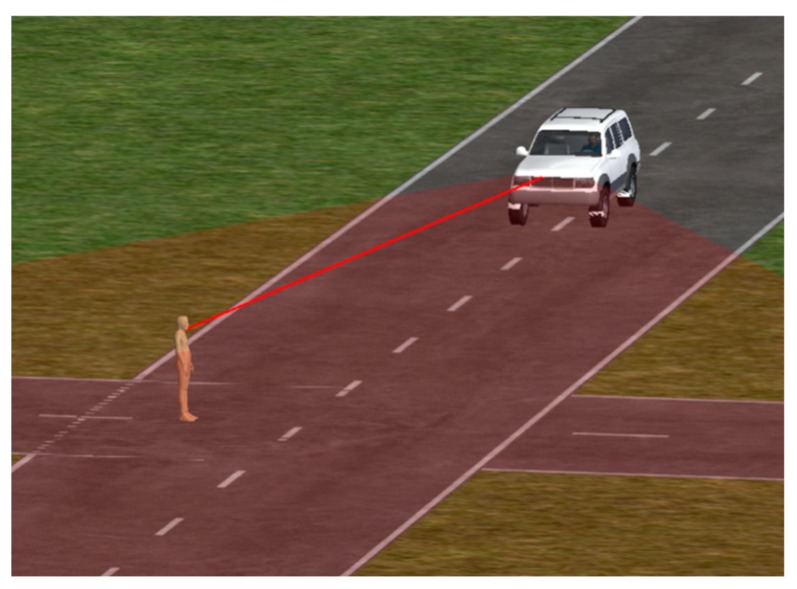
The AEB-P system pedestrian test scenarios.

A straight-line asphalt or concrete urban road with the east-west direction was established. The design slope of the road was 0, the road surface’s sliding adhesion coefficient was 0.75, and the peak adhesion coefficient was 0.95. The north-south straight-line urban road with the same characteristics was also established, and the crossroad model was used to simulate the pedestrian crossing road test scene. The long-distance millimeter-wave radar was mounted behind the grille in the middle of the vehicle’s head, and the mid-range millimeter-wave radar was mounted on both sides of the vehicle front. We set the azimuth of the long-distance millimeter-wave radar to ±10°; the maximum detection distance was 100 m, and only one radar was used. The azimuth of the medium-distance millimeter-wave radar was set to ±45°; the maximum detection distance was 50 m, and two medium-distance millimeter-wave radars were used. The CVFA and CVNA pedestrian test scenarios of the AEB-P were established, respectively, and the simulation results are shown in Figure 24.

### 5.3. Analysis of Joint Simulation Results of AEB-P System

**Figure 25:**
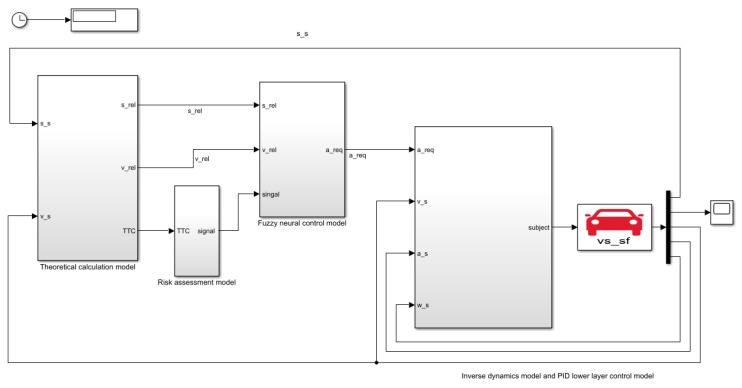
The AEB-P joint simulation system.

The co-simulation was performed using CarSim and Simulink simulation software, and the established AEB-P joint simulation system is shown in Figure 25. The AEB-P co-simulation system was divided into five parts, namely: (1) CarSim dynamic model of a certain E-class SUV, including braking system, transmission system, tire model, suspension model, etc.; (2) PID lower control model and inverse dynamic model; (3) decision-making layer of the AEB-P system-fuzzy neural network control model; (4) risk assessment model; (5) theoretical calculation model that was mainly responsible for analyzing the driving information collected by radar and analyzing the data through the theoretical calculation, and filtering out the information required by the AEB-P system.

The requirements for the AEB-P system in the simulation test were as follows. Under the condition of normal driving behaviors and driving environment, when there was no potential pedestrian collision, the AEB-P system should not interfere with a driver and should not issue any warning and braking signals. In the event of a potential collision hazard to pedestrians, the hazard should be detected in time, and the level of danger should be classified and an alarm should beep to alert a driver that there might be a pedestrian collision hazard; if the driver failed to take appropriate braking measures, the AEB-P system should promptly intervene to control the vehicle by using the deceleration to avoid collision or to reduce the impact of pedestrian collision damage. To make the AEB-P system better, it was required not only to avoid pedestrians but also to ensure a certain safety distance so that pedestrians would not be scared. Taking the urban road intersection as a test scenario, the simulation analysis was conducted, and the AEB-P system verification and evaluation were performed according to the simulation analysis results.

#### 5.3.1. CVFA-25Pedestrian Test Scenario

**Figure 26:**
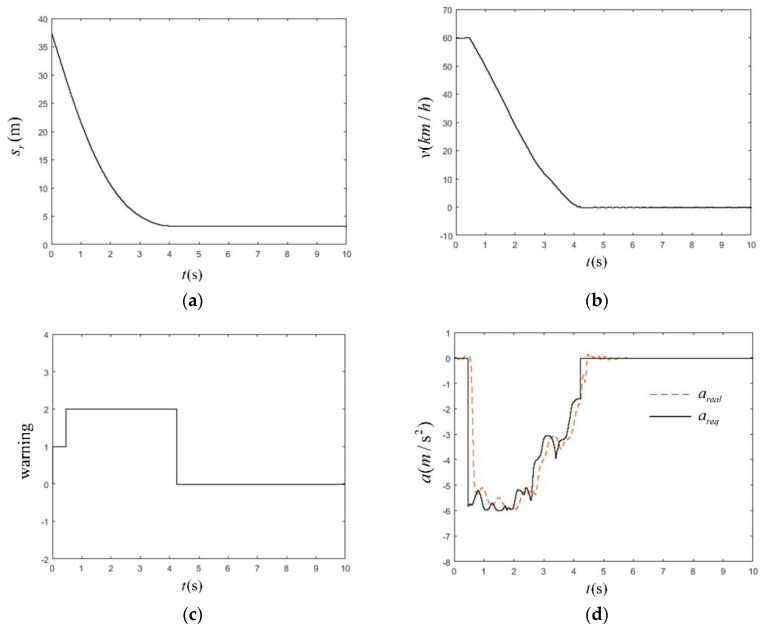
The simulation time as a function of station (**a**), velocity (**b**), warning signal (**c**), deceleration of the vehicle (**d**) in CVFA-25 scenario under high-speed working conditions.

This test scenario mainly simulated urban road traffic conditions where a pedestrian traversed the road from the far side of the road where a vehicle was traveling. If a vehicle did not take the corresponding braking measures, it would have a 25% lateral deviation from the pedestrian crossing collision. The working conditions are shown in Table 10. The simulation results at high speed (60 km/h) are shown in Figure 26.

In Figure 26a, the minimum relative longitudinal distance between $s_{r}$ and pedestrian after 3.3 s was 3.3 m, and the braking distance was 26.67 m. In Figure 26b, the vehicle speed was 60 km/h during the first 0.5 s. The vehicles were first driving at a constant speed, and then started braking; the braking time lasted for 3.6 s. In Figure 26c, because the test condition was the most dangerous working condition in the CVFA-25 test scenario, in order to ensure the braking effect, the warning time was sacrificed and only lasted 0.5 s, the AEB-P system was released after 4.1 s, and there were no accidents or leaks during the whole process. In Figure 26d, the AEB-P system expected the deceleration *a_req_* to be −6 m/s^2^, and the actual deceleration could be timely. In response to the required deceleration value, the maximum delay of the system was 0.286 s, and the maximum errors—*a_real_* and *a_req_*—in steady-state phases were only 0.23 m/s^2^. After the danger was released, the brakes were withdrawn in time, and there was no interference with the normal operation of a driver.

#### 5.3.2. CVFA-50Pedestrian Test Scenario

**Figure 27:**
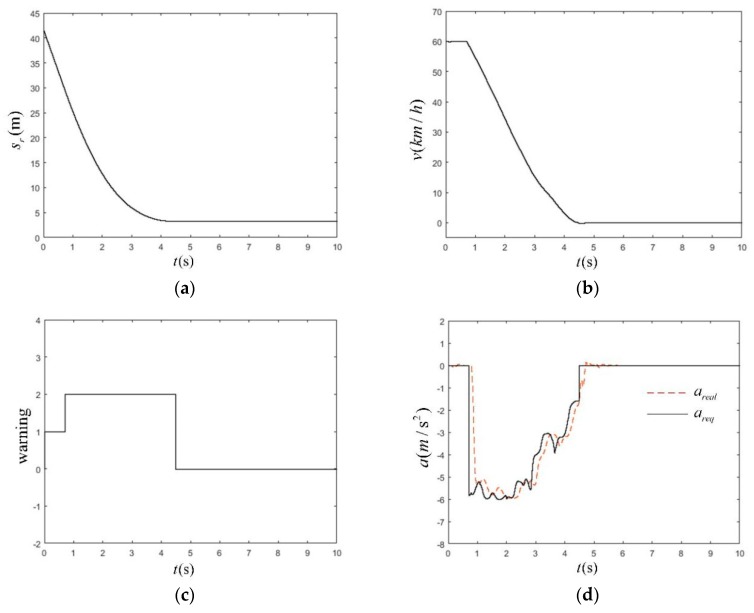
The simulation time as a function of station (**a**), velocity (**b**), warning signal (**c**), deceleration of the vehicle (**d**) in CVFA-50 scenario under high-speed working conditions.

The test conditions mainly simulated the urban road traffic conditions where pedestrians traversed the road from the far side of the road where a vehicle was traveling. If a vehicle did not take the corresponding braking measures, it would have a 50% side collision with the pedestrian crossing. The working conditions are given in Table 11. The simulation results of typical operating conditions, i.e., at a high speed of 60 km/h are shown in Figure 27.

In Figure 27a, it can be seen that the minimum relative longitudinal distance from a pedestrian after a vehicle was passing for 4.3 s was 3.3 m, and the braking distance was 26.66 m. As shown in Figure 27b, the vehicle started to apply the brake at 0.7 s, and the braking time lasted for 3.6 s. In Figure 27c, the AEB-P system sent out an early warning signal at the beginning of the test, the warning time was 0.7 s, and there was no false alarm or missed alarm in the whole process. Lastly, in Figure 27d, the AEB-P system expected the deceleration *a_req_* to be from −5.2 m/s^2^ to −6 m/s^2^, the actual deceleration could respond in time, and *a_real_* and *a_req_* errors in the steady-state phase were small. After the danger was released, the brakes were withdrawn in time, and there was no interference with the normal operation of a driver.

#### 5.3.3. CVNA-25 Near-End Pedestrian Test Scenario

The conditions of this test mainly simulated urban road traffic conditions where pedestrians traversed the road from the near side of the vehicle’s road. If a vehicle did not take the corresponding braking measures, it would have a 25% side collision with the pedestrian crossing. This test scenario is the most dangerous of all the scenarios, and the design conditions are shown in Table 12.

Based on the CVFA test scenario results, it can be concluded that the test conditions of the same initial vehicle speed and the control strategy performed by the AEB-P system were similar, which provided a more comprehensive analysis of the control strategies and functions of the AEB-P system under different test conditions. In the CVNA scenario, the initial vehicle speed was selected to be 30 km/h and 50 km/h, respectively. The simulation results of the CVNA-25 test scenario are shown in Figure 27. 

**Figure 28:**
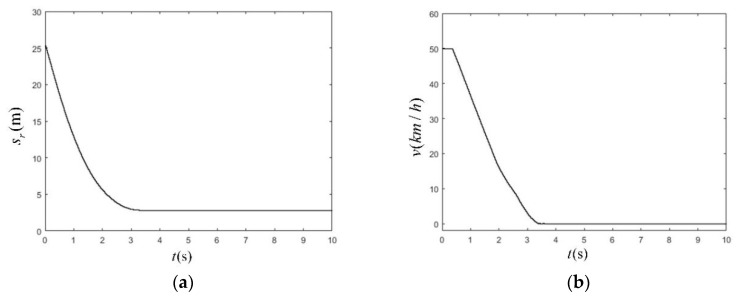

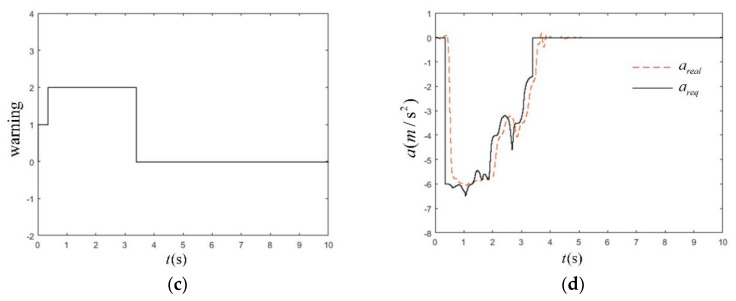
The simulation time as a function of station (**a**), velocity (**b**), warning signal (**c**), deceleration of the vehicle (**d**) in CVNA-25 scenario at the vehicle speed of 50 km/h.

As shown in Figure 28a, at 3.46 s, the vehicle completed braking successfully and stopped timely. The minimum longitudinal relative distance from the pedestrian was 2.86 m, and the braking distance was 18.03 m. In Figure 28b, the vehicle started to apply the brake at 0.34 s, and the braking process lasted for 12 s. In Figure 28c, the AEB-P warning system sent a pedestrian collision warning signal at the beginning of the test. The alarm duration was 0.34 s, indicating that the working condition was dangerous, and the collision risk was very high. After that, the AEB-P warning system issued an emergency braking signal. At 2.46 s, the brake was released, and no alarm or false alarm signal appeared in the entire early warning braking process. In Figure 28d, the actual braking deceleration of the vehicle and the expected braking deceleration coincided well. The maximum delay time was 0.35 s. The deceleration curve changed smoothly. When the *a_req_* dynamic output was stable, *a_real_* and *a_req_* errors were 0.21 m/s^2^. At the beginning of the braking process, the expected deceleration was about −6 m/s^2^, further indicating that the risk of a collision was higher. The AEB-P system reduced the expected deceleration to the range (−3.92 to −3.20 m/s^2^) at the end of the braking phase. The AEB-P system did not interfere with the normal operation of the driver during the entire warning and braking process.

#### 5.3.4. CVNA-75 Near-End Pedestrian Test Scenario

**Figure 29:**
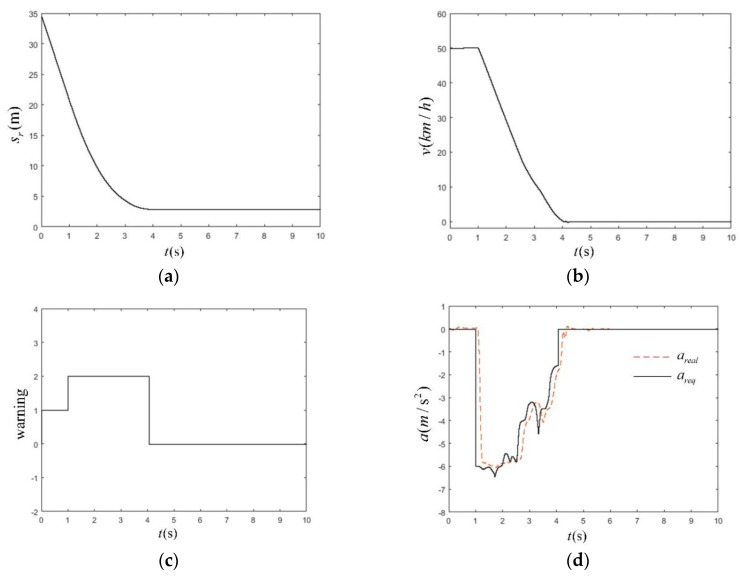
The simulation time as a function of station (**a**), velocity (**b**), warning signal (**c**), deceleration of the vehicle (**d**) in CVNA-75 scenario at the vehicle speed of 50 km/h.

The conditions of this test mainly simulated urban road traffic conditions where pedestrians traversed the road from the near side of the vehicle’s road. If a vehicle did not take the corresponding braking measures, it would have a 75% side collision with the pedestrian crossing. The simulation parameters are shown in Table 13. The initial vehicle speed was selected to be 30 km/h and 50 km/h, respectively. The simulation results of the CVNA-75 are shown in Figure 28. In the CVNA-75 test at the initial vehicle speed of 50 km/h, as shown in Figure 29a, at 4.04 s, the vehicle completed braking and stopped, the minimum longitudinal relative distance from the pedestrian was 2.88 m, and the braking distance was 18.05 m. In Figure 29b, at the initial vehicle speed of 50 km/h, the braking started at 0.98 s, and the braking process lasted for 3.06 s. In Figure 29c, the AEB-P warning system sent a pedestrian collision warning signal at the beginning of the test. The alarm duration was 0.98 s. After that, the AEB-P system sent an emergency brake signal. At 4.04 s, the brake was released. There was no leakage alarm or false alarm signal during the whole early warning braking process. As shown in Figure 29d, the actual vehicle deceleration and expected braking deceleration coincided well, the deceleration fluctuation was small, the maximum error of *a_real_* and *a_req_* steady-state was 0.17 m/s^2^, the expected deceleration was about −6 m/s^2^ at the beginning of the braking process, the risk of a collision was high, and the AEB-P system was about to end in braking. The stage reduced the expected deceleration to the range (−3.99 to −10 m/s^2^). The entire warning and braking process of the AEB-P system did not interfere with the normal operation of the driver.

The test simulation results of the CVFA-25, CVFA-50, CVNA-25, and CVNA-75 scenarios are summarized in Table 14.

## 6. Conclusions

In this paper, an automatic emergency braking pedestrian (AEB-P) longitudinal active collision avoidance system was proposed, and the following research conclusions were drawn.

(1) Combining the characteristics and requirements of the AEB-P pedestrian test conditions, the TTC risk assessment model integrating the brake safety distance theory was established, and the AEB-P system classification warning mechanism was proposed. The pedestrian collision risk degree was graded and determined. The TTC values and the corresponding safety levels under different vehicle speed conditions were defined.

(2) The proposed system was verified by simulation tests. In the CVFA test scenario, the vehicle did not collide with pedestrians, ensuring the safety of pedestrians. The redundant safety distance between vehicles and pedestrians was 2.08–3.3 m. During the braking process, the vehicle speed curve changed smoothly, and the AEB-P warning system could accurately predict the collision risk and reasonably classify the safety level. The warning period was 0.5–1.5 s, and it did not interfere with the normal operation of the driver, and there was no false alarm or leaking alarm. The actual acceleration curve and the expected acceleration curve coincided well, and the error was within a reasonable range. The braking strength was 4.5–6.1 m/s^2^. The braking distance was 3.46–26.67 m. The results showed that the AEB-P system could adjust the braking force in real-time according to the degree of danger, reducing the discomfort caused by emergency braking to the driver and passengers.

(3) In the CVNA test scenario, the AEB-P system also implemented the pedestrian emergency collision avoidance function, which was consistent with the CVFA test results. In the CVNA-25 scenario, when the vehicle speed was 60 km/h, the AEB-P system considered the pedestrian safety as the highest priority, sacrificing the collision warning time, directly intervening in the vehicle brake control, and achieving effective avoidance of collision with pedestrians. 

The obtained results show that the control strategy proposed in this paper is accurate and reliable, and can flexibly allocate early warning and braking time according to the changes in actual working conditions, and reduce the occurrence of pedestrian collision accidents.

In the future work, the research of automatic emergency braking in complex scenarios will be further studied, and the factors, such as weather, road, vehicle state, and driver behavior, will be fully considered. In addition, we will also study the reliability of the control model with the traffic participants, such as bicycles, motorcycles, tricycles, and other vehicles, with Chinese characteristics, and verify the reliability of the proposed simulation model through the real vehicle test.

## Figures and Tables

**Table 1:** Main vehicle parameters.

Vehicle Parameter	Value	Unit
Engine rated power	125	kw
Maximum engine torque	251	N·m
Vehicle curb quality	1615	kg
Air resistance coefficient	0.32	/
Frontal area	2.73	m^2^
Rolling friction coefficient	0.004	/
Wheelbase	2750	mm
Tread	1615	mm
The center of gravity to the center of the front wheel	1107	mm
Height of gravitational center	780	mm
Main reducer reduction ratio	3.48	/
Tire rolling radius	0.3588	mm
Powertrain transmission efficiency	0.9	/
Crankshaft moment of inertia	0.16	kg·m^2^
Engine idle speed	750	r/min
Transmission shift time	0.5	S
Transmission 1 speed ratio	3.917	/
Transmission 2 speed ratio	2.042	/
Transmission 3 speed ratio	1.257	/
Transmission 4 speed ratio	0.909	/
Transmission 5 speed ratio	0.902	/
Transmission 6 speed ratio	0.773	/
Transmission reverse speed ratio	−4.298	/
Unsprung mass of front suspension	122.8	kg
Unsprung mass of rear suspension	111	kg
Wheel slip ratio with ABS (anti-lock brake system) system	10~30	%
Brake disc quality	9.65	kg
Brake pedal lever ratio	3.6	/
Master cylinder diameter	25.4	mm
Brake disk specific heat capacity at 0 celsius degree	1.0425	kJ/kg·°C
The pressure generated by unit flow in brake caliper hydraulic cylinder	4.10 × 10^−6^	MPa/(mm^3^/s)
Time delay for starting boost	0.001	S
Closed time delay	0.001	S
Tire size	265/75R16	mm
Tire stiffness	502	N/mm
Tire vertical load	11,500	N
Tire maximum load	100,000	N
Effective rolling radius	393	mm
Free radius	402	mm

**Table 2:** The safety rating and TTC (time to collision) range under different working conditions.

Vehicle Speed (km/h)	TTC Value Range (s)	Security Level
20	0~1	III
	1~2.5	II
	>2.5	I
30	0~1.1	III
	1.1~2.6	II
	>2.6	I
40	0~1.3	III
	1.3~2.8	II
	>2.8	I
50	0~1.5	III
	1.5~3	II
	>3	I
60	0~1.8	III
	1.8~3.3	II
	>3.3	I

**Table 3:** The learning parameters of the longitudinal relative distance.

		Z0	P1	P2	P3	P4	P5	P6	P7	P8
Δs	c	0	0.5	1	1.5	2	2.67	3.43	4.27	5
	$\sigma^{\prime }$	0.33	0.37	0.35	0.3	0.41	0.5	0.66	0.55	0.54
	$\sigma^{\prime \prime }$	0.35	0.33	0.34	0.37	0.42	0.58	0.71	0.66	0.62

**Table 4:** The learning parameters of the longitudinal relative velocity.

		N11	N10	N9	N8	N7	N6	N5	N4	N3	N2	N1	Z0
$v_{r}$	c	−8	−6.9	−6	−5.1	−4	−3.2	−2.5	−2	−1.5	−1	−0.5	0
	$\sigma^{\prime }$	0.8	−0.85	0.77	0.8	0.59	0.52	0.43	0.3	0.33	0.35	0.31	0.36
	$\sigma^{\prime \prime }$	0.7	0.79	0.7	0.79	0.59	0.53	0.4	0.33	0.35	0.36	0.39	0.28

**Table 5:** The learning parameters of the expected deceleration.

		N9	N8	N7	N6	N5	N4	N3	N2	N1	Z0
$a_{r}$	c	−1	−0.9	−0.8	−0.7	−0.6	−0.5	−0.4	−0.3	−0.16	0
	$\sigma^{\prime }$	0.06	0.08	0.06	0.08	0.05	0.07	0.08	0.08	0.1	0.12
	$\sigma^{\prime \prime }$	0.06	0.08	0.06	0.08	0.07	0.07	0.07	0.08	0.11	0.11

**Table 6:** The fuzzy control rules.

$a_{r}$	$v_{r}$											
Δs	**Z0**	**N1**	**N2**	**N3**	**N4**	**N5**	**N6**	**N7**	**N8**	**N9**	N10	N11
Z0	N1	N5	N7	N8	N9	N9	N9	N9	N9	N9	N9	N9
P1	Z0	N2	N2	N3	N5	N7	N7	N9	N9	N9	N9	N9
P2	Z0	N1	N1	N3	N3	N4	N5	N7	N7	N7	N7	N9
P3	Z0	N1	N1	N2	N2	N3	N4	N5	N6	N6	N7	N8
P4	Z0	Z0	N1	N1	N2	N2	N3	N4	N5	N5	N6	N7
P5	Z0	Z0	Z0	N1	N1	N1	N2	N3	N3	N5	N5	N6
P6	Z0	Z0	Z0	Z0	N1	N1	N1	N3	N3	N5	N5	N6
P7	Z0	Z0	Z0	Z0	Z0	Z0	Z0	N2	N2	N4	N5	N5
P8	Z0	Z0	Z0	Z0	Z0	Z0	Z0	N1	N2	N6	N7	N8

**Table 7:** Partial training data.

$a_{r}$	$v_{r}$											
Δs	**0**	**−1**	**−2**	**−3**	**−3.5**	**−4**	**−4.5**	**−5**	**−5.5**	**−6**	−7	−8
0	−0.16	−0.8	−0.97	−0.97	−0.97	−0.98	−0.98	−0.98	−0.98	−0.98	−0.98	−0.98
0.5	−0.16	−0.3	−0.6	−0.6	−0.81	−0.89	−0.89	−0.97	−0.97	−0.97	−0.97	−0.97
1	−0.06	−0.16	−0.4	−0.4	−0.67	−0.81	−0.81	−0.81	−0.85	−0.97	−0.97	−0.97
1.5	−0.04	−0.16	−0.3	−0.3	−0.53	−0.67	−0.67	−0.7	−0.7	−0.7	−0.81	−0.98
2	−0.04	−0.16	−0.3	−0.3	−0.43	−0.56	−0.56	−0.6	−0.64	−0.7	−0.8	−0.9
2.5	−0.04	−0.05	−0.16	−0.16	−0.32	−0.46	−0.46	−0.5	−0.54	−0.6	−0.7	−0.81
3	−0.06	−0.06	−0.16	−0.16	−0.26	−0.45	−0.45	−0.45	−0.5	−0.58	−0.7	−0.81
3.5	−0.04	−0.04	−0.16	−0.16	−0.21	−0.40	−0.4	−0.4	−0.47	−0.6	−0.7	−0.81
4	−0.04	−0.04	−0.07	−0.07	−0.15	−0.31	−0.31	−0.3	−0.39	−0.58	−0.61	−0.71
4.5	−0.04	−0.04	−0.04	−0.04	−0.14	−0.25	−0.25	−0.3	−0.4	−0.57	−0.60	−0.69
5	−0.04	−0.04	−0.04	−0.04	−0.1	−0.23	−0.23	−0.3	−0.36	−0.49	−0.6	−0.6

**Table 8:** PID (proportional integral derivative) values under different working conditions.

Vehicle Speed (km/h)	$K_{P}$	$T_{I}$	$T_{D}$
20	4	100	0
30	4	20	0
40	4	30	0
50	4	35	0
60	4	25	0

**Table 9:** Autonomous emergency braking pedestrian (AEB-P) system test scenarios.

Test Scenario	Pedestrian Speed (km/h)	Vehicle Speed (km/h)
CVFA (Car-to-VRU Farside Adult)-25	6.5	20–60
CVFA-50	6.5	20–60
CVNA (Car-to-VRU Farside Adult)-25	5	20–60
CVNA-75	5	20–60

**Table 10:** CVFA-25 simulation conditions.

Initial Vehicle Speed (km/h)	Longitudinal Initial Distance of the Vehicle Relative to the Pedestrian (m)	Pedestrian Walking Speed (km/h)
20	12.446	6.5
30	18.669	6.5
40	24.890	6.5
50	31.115	6.5
60	37.338	6.5

**Table 11:** The CVFA-50 simulation conditions.

Initial Vehicle Speed (km/h)	Longitudinal Initial Distance of a Vehicle Relative to a Pedestrian(m)	Pedestrian Walking Speed (km/h)
20	13.846	6.5
30	20.7692	6.5
40	27.692	6.5
50	34.6153	6.5
60	41.5383	6.5

**Table 12:** The CVNA (Car-to-VRU Farside Adult)-25 simulation conditions.

Initial Vehicle Speed (km/h)	Longitudinal Initial Distance of the Vehicle Relative to the Pedestrian (m)	Pedestrian Walking Speed (km/h)
20	10.18	5
30	15.27	5
40	20.36	5
50	25.45	5
60	30.54	5

**Table 13:** The CVNA (Car-to-VRU Farside Adult)-75 simulation conditions.

Initial Vehicle Speed (km/h)	Longitudinal Initial Distance of the Vehicle Relative to the Pedestrian (m)	Pedestrian Walking Speed (km/h)
20	13.82	5
30	20.73	5
40	27.64	5
50	34.55	5
60	41.46	5

**Table 14:** Simulation results under the AEB-P test conditions.

Speed (km/h)	Testing Scenario	Pedestrian Speed (km/h)	Alarm Duration (s)	Brake Duration (s)	Braking Distance (m)	False, Missed Alarms	Maximum Brake Deceleration (m/s^2^)	Brake-Stop Relative Distance (m)	Collision Occurred
20	CVFA-25	6.5	1.25	1.75	3.48	0	−4.5	2.08	NO
	CVFA-50	6.5	1.5	1.5	3.46	0	−4.6	2.08	NO
	CVNA-25	5	0.83	1.55	3.47	0	−4.5	2.08	NO
	CVNA-75	5	1.5	1.5	3.47	0	−4.62	2.1	NO
30	CVFA-25	6.5	1.1	2.04	7.14	0	−5.9	2.17	NO
	CVFA-50	6.5	1.36	1.97	7.16	0	−6.1	2.18	NO
	CVNA-25	5	0.74	2.11	7.1	0	−6.19	2.14	NO
	CVNA-75	5	1.34	2.06	7.15	0	−6	2.18	NO
40	CVFA-25	6.5	1	2.65	11.86	0	−6	2.608	NO
	CVFA-50	6.5	1.2	2.67	11.86	0	−6.1	2.65	NO
	CVNA-25	5	0.53	2.52	11.84	0	−6.11	2.6	NO
	CVNA-75	5	1.19	2.58	11.88	0	−6.1	2.61	NO
50	CVFA-25	6.5	0.74	3.07	17.94	0	−6	2.9	NO
	CVFA-50	6.5	0.97	3.08	17.99	0	−5.97	2.86	NO
	CVNA-25	5	0.34	12	18.03	0	−6.02	2.86	NO
	CVNA-75	5	0.97	3.06	18.05	0	−6.1	2.9	NO
60	CVFA-25	6.5	3.6	3.6	26.67	0	−6	3.3	NO
	CVFA-50	6.5	3.6	3.6	26.66	0	−6	3.3	NO
	CVNA-25	5	3.78	3.78	27.24	0	−6	3.3	NO
	CVNA-75	5	3.77	3.77	26.74	0	−6	3.3	NO
